# Development of a leucine-rich repeat-containing protein 15-targeted radio-immunotheranostic approach to deplete pro-tumorigenic mechanisms and immunotherapy resistance

**DOI:** 10.1038/s41392-025-02410-9

**Published:** 2025-09-30

**Authors:** Claire M. Storey, Mohamed Altai, Katharina Lückerath, Wahed Zedan, Henan Zhu, Lara Breuer, Marija Trajkovic-Arsic, Julie Park, Abbie Hasson, Jens Siveke, Diane Abou, Haley Marks, Enna Ulmert, Alexander Ridley, Marcella Safi, Urpo Lamminmäki, Constance Yuen, Susanne Geres, Liqun Mao, Michael Cheng, Sumit K. Subudhi, Bilal A. Siddiqui, Noah Federman, Johannes Czernin, Ken Herrmann, Laurent Bentolila, Xia Yang, Thomas G. Graeber, Robert Damoiseaux, Daniel Thorek, David Ulmert

**Affiliations:** 1 Department of Molecular & Medical Pharmacology, University of California Los Angeles (UCLA), Los Angeles, CA USA; 2 Division of Oncology and Pathology, Department of Clinical Sciences, Lund University, Lund, Sweden; 3 Lund University Cancer Centre (LUCC), Lund University, Lund, Sweden; 4 Department of Nuclear Medicine, University Hospital Essen, University of Duisburg-Essen, DKTK, Essen, Germany; 5 Bridge Institute of Experimental Tumor Therapy (BIT) and Division of Solid Tumor Translational Oncology (DKTK), West German Cancer Center, University Hospital Essen, University of Duisburg-Essen, Essen, Germany; 6 German Cancer Consortium (DKTK), partner site Essen, a partnership between German Cancer Research Center (DKFZ) and University Hospital Essen, Heidelberg, Germany; 7 Department of Radiology, Washington University School of Medicine, St. Louis, MO USA; 8 Siteman Cancer Center, Washington University School of Medicine, St. Louis, MO USA; 9 Advanced Light Microscopy and Spectroscopy Laboratory, California Nanosystems Institute, UCLA, Los Angeles, CA USA; 10 Department of Life Technologies, University of Turku, Turku, Finland; 11 California NanoSystems Institute, UCLA, Los Angeles, CA USA; 12 Bioinformatics Interdepartmental Program, University of California, Los Angeles, CA USA; 13 Department of Genitourinary Medical Oncology, The University of Texas MD Anderson Cancer Center, Houston, TX USA; 14 Pediatric Bone and Soft Tissue Sarcoma Program at UCLA, Los Angeles, CA USA; 15 Ahmanson Translational Theranostics Division, Department of Molecular and Medical Pharmacology, UCLA, Los Angeles, CA USA; 16 Department of Integrative Biology & Physiology, University of California, Los Angeles, Los Angeles, CA USA; 17 UCLA Metabolomics Center, University of California, Los Angeles (UCLA), Los Angeles, CA USA; 18 Department of Biomedical Engineering, Washington University in St. Louis, St. Louis, MO USA; 19 Department of Urology, Institute of Urologic Oncology, UCLA, Los Angeles, CA USA; 20 Jonsson Comprehensive Cancer Center, David Geffen School of Medicine, UCLA, Los Angeles, CA USA; 21 Eli and Edythe Broad Center of Regenerative Medicine and Stem Cell Research, UCLA, Los Angeles, CA USA

**Keywords:** Drug development, Tumour biomarkers

## Abstract

Leucine-rich repeat containing 15 (LRRC15) has emerged as an attractive biomarker and target for cancer therapy. Transforming growth factor-β (TGFβ) induces the expression of this plasma membrane protein specifically in aggressive and treatment resistant tumor cells derived from mesenchymal stem cells, with minimal expression observed in non-neoplastic tissues. We have developed a humanized monoclonal antibody, DUNP19, that specifically binds with high affinity to a phylogenetically conserved LRRC15 epitope and is rapidly internalized upon LRRC15 binding. In multiple subcutaneous and orthotopic tumor xenograft mouse models, Lutetium-177 labeled DUNP19 ([^177^Lu]Lu-DUNP19) enabled non-invasive imaging and molecularly precise radiotherapy to LRRC15-expressing cancer cells and murine cancer-associated fibroblasts, effectively halting tumor progression and prolonging survival with minimal toxicity. Transcriptomic analyses of [^177^Lu]Lu-DUNP19-treated tumors reveal a loss of pro-tumorigenic mechanisms, including a previously reported TGFβ-induced LRRC15+ signature associated with immunotherapy resistance. In a syngeneic tumor model, administration of [^177^Lu]Lu-DUNP19 significantly potentiated checkpoint-blockade therapy, yielding durable complete responses. Together, these results demonstrate that radio-theranostic targeting of LRRC15 with DUNP19 is a compelling precision medicine platform for image-guided diagnosis, eradication, and reprogramming of LRRC15+ tumor tissue that drives immuno-resistance and disease aggressiveness in a wide range of currently untreatable malignancies.

## Introduction

Advancements in the field of immuno-engineering have catalyzed the development of potent antibody-based treatments, the largest class of biologic cancer treatments. However, these therapies are currently applicable towards a narrow subset of malignancies, with aberrantly high expression and signaling pathways.^1^ In particular, antibody-based therapeutics that engage the immune system through immune checkpoint inhibition (ICI) or bispecific engagement of T cells have had a significant impact in recent years on both clinical management and pharmaceutical drug development strategy. These therapeutics seek to engage proteins on cancer and immune cells in order for the patients’ own immune system to recognize, seek out, and destroy malignant cells. A persistent challenge remains in identifying widely expressed surface antigens for therapy-resistant and metastasized solid tumors.^2^ The integration of antibodies targeting these biomarkers, alongside advancements in radiochemistry and non-invasive imaging technologies for visualization of radiolabeled antibody distribution, holds substantial promise. This synergy provides the basis for radio-theranostics; selecting patients via imaging that could benefit from treatment and using the same antibody armed with cytotoxic radionuclides for therapy.^3^ Such an approach could revolutionize cancer treatment, expanding its reach to address a broader spectrum of therapy-resistant and disseminated cancers.

Leucine-Rich Repeat Containing 15 (LRRC15) is a transmembrane protein expressed in TGFβ-driven cancer-associated fibroblasts (CAFs) and cancer cells of mesenchymal stem cell origin, including sarcomas and glioblastomas.^4,5^ While there are few studies investigating the exact role of LRRC15 in cancer pathobiology, recent evidence suggests a role in Wnt/β-catenin signaling pathway activation to promote invasion and metastasis.^6,7^ In tumor tissue, the presence of LRRC15+ cells correlated with resistance to immune checkpoint blockade, increased risk for metastasis, and lower survival rates in pancreatic cancer mouse models and retrospective patient cohorts, underscoring LRRC15 role as an immunomodulator governed by the TGFβ pathway.^4,8,9^ Notably, LRRC15 has little or no expression in healthy tissue, making the protein a highly promising target for therapeutic intervention.^5^

Here, we describe the development of a highly specific monoclonal antibody (mAb) targeting LRRC15 (designated as DUNP19) that exhibits high specificity for a phylogenetically conserved epitope present on both human and murine LRRC15, and, upon binding to target cells, rapidly internalizes. We exploit DUNP19’s rapid internalization profile by labeling the mAb with both diagnostic and cytotoxic radionuclides, transforming it into a dual-purpose agent for use in non-invasive imaging and in therapeutic applications. The potential of this technology extends to personalized treatment strategies and dose planning, maximizing the therapeutic index for individual patients. A central design choice in the development of this radioimmunotheranostic platform was the use of a full-length IgG1 antibody format for DUNP19. Full-sized mAbs offer several key advantages that align with the demands of both diagnostic and therapeutic applications. These include high binding affinity and exceptional in vivo stability, both of which are essential for sustained target engagement and effective tumor accumulation of radiolabeled compounds. The longer half-life of full-sized mAbs—conferred by FcRn-mediated recycling—facilitates high tumor retention, which is crucial for therapeutic radionuclides with multi-day half-lives, such as Lutetium-177. While smaller antibody formats such as fragments or nanobodies offer faster systemic clearance and reduced bone marrow exposure, these formats generally suffer from lower binding avidity, increased renal uptake, and limited ability to mediate immune effector functions. Furthermore, the well-established manufacturing protocols and regulatory pathways for full-length IgG1 antibodies streamline clinical translation, whereas fragments often require extensive re-engineering and individualized optimization. Importantly, the IgG1 framework used here also allows for future therapeutic adaptability, such as conversion into antibody-drug conjugates or bispecific constructs, without redesigning the antigen-binding domain.

To address the challenge of target heterogeneity that limits the efficacy of classical antibody-drug conjugates, we deployed this novel platform to harness the radiation deposited in LRRC15+ cells to eliminate adjacent LRRC15-null tumor tissue. This approach utilizes Lutetium-177, a clinically relevant beta particle emitter with a half-life of 6.7 days and a maximum tissue penetration of 1.5 mm. As a beta-particle emitter, Lutetium-177 has the potential to cause DNA strand breaks across a span of 10–50 cell diameters. This extended path length enables a crossfire effect, delivering ionizing radiation to both target and nearby non-target cells^5,10^ For diagnostic purposes, we also functionalized DUNP19 with positron-emitting Copper-64 ([^64^Cu]Cu-DUNP19). Importantly, systemic treatment with [^177^Lu]Lu-DUNP19 resulted in significant therapeutic efficacy and survival in tumor-bearing mice. In addition, transcriptomic profiling of [^177^Lu]Lu-DUNP19-treated tissues revealed progressive loss of TGFβ-driven genomic signatures associated with malignant disease aggressiveness and immunotherapy resistance. Synergy of [^177^Lu]Lu-DUNP19 with immune checkpoint inhibition validated the functional relevance of these data. Taken together, these findings highlight the potential of DUNP19 as a radio-theranostic modality for non-invasive detection, targeting, and reprogramming of immuno-suppressive gene signatures within cancer cells and the tumor microenvironment.

## Results

### LRRC15 can be targeted utilizing the mAb DUNP19

**Fig. 1:**
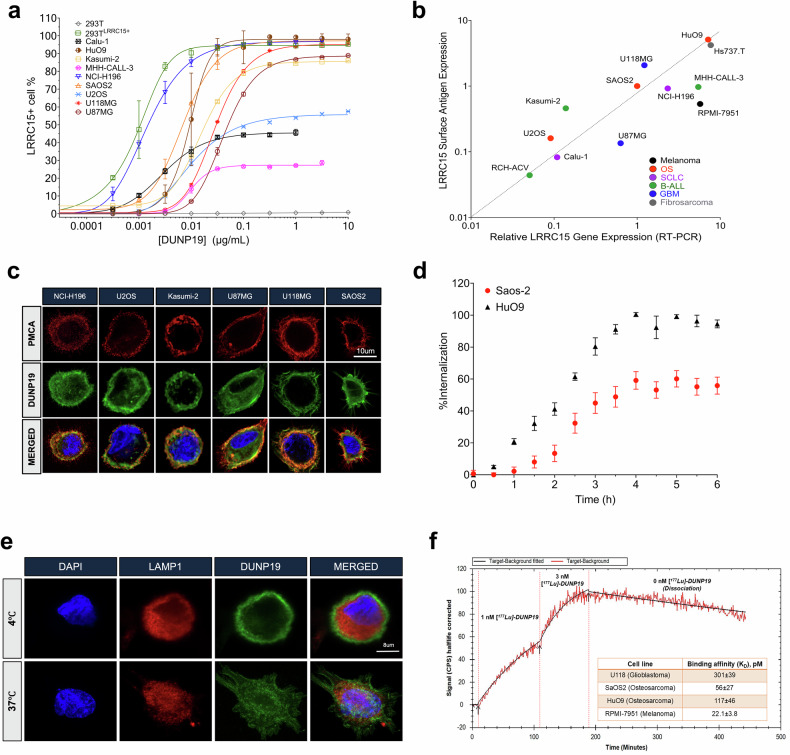
In vitro evaluation of DUNP19’s binding profile to LRRC15. **a** Flow cytometry demonstrates that DUNP19 binds with picomolar affinity to LRRC15 molecules on various cell lines (*n* = 3–6 per cell line), exhibiting distinct expression levels and tissue origins. Detection of LRRC15 was based on the assessment of antigen-antibody equilibrium. **b** Analysis across a diverse range of cell lines (*n* = 3–6 per cell line) unveils correlations between LRRC15 mRNA expression and the abundance of LRRC15 molecules bound by DUNP19. **c** Confocal microscopy of various cell lines incubated with AlexaFluor647-labeled DUNP19 at room temperature, followed by staining for plasma membrane-associated calcium ATPase (PCMA) and DNA (DAPI). Images reveal that DUNP19 binding corresponds with LRRC15 expression levels and co-localizes with LRRC15 (*n* = 3/group). **d** Internalization rates of AlexaFluor647-labeled DUNP19 at 37 °C were examined in LRRC15-expressing HuO9 (*n* = 12) and SAOS2 (*n* = 12) cells using live confocal microscopy. The endocytic process of DUNP19 is accelerated in cells with higher LRRC15 abundance. **e** Confocal microscopy of SAOS2 cells incubated with AlexaFluor647-labeled DUNP19 at 4 °C or 37 °C, co-stained for lysosomes (LAMP1) and DNA (DAPI). DUNP19 is exclusively found in the plasma membrane at the lower temperature demonstrating that the rapid endocytosis after binding to LRRC15 is an active, energy-requiring process (one representative experiment out of 3 is shown). **f** LigandTracer sensorgram of [177Lu]Lu-DUNP19 binding to LRRC15-expressing HuO9 cells, measured at 1 nM and 3 nM concentrations. Cell-bound activity, presented as CPS, was used to determine association, dissociation rates, and equilibrium dissociation constants (K_D_). The table shows estimated K_D_ values for various LRRC15-expressing cell lines

Our initial investigations tested the specificity of DUNP19 and demonstrated binding to both human and murine recombinant LRRC15 with high affinity (Supplementary Fig. 1a, Supplementary Fig. 1b). DUNP19 exhibited a K_D_ of 175 pM for human LRRC15 and 370 nM for murine LRRC15 (Supplementary Fig. 1b, Supplementary Materials); this difference is attributable to species-specific epitope variations in LRRC15’s extracellular domain. Specific interaction of DUNP19 with LRRC15 was further substantiated by immunoprecipitation of the LRRC15 protein from U118MG cell lysates with DUNP19-coated magnetic beads (Supplementary Fig. 1c). We next characterized the binding of DUNP19 to LRRC15 across a wide array of cancer cell lines from a range of lineages including melanoma (RPMI7951), glioblastoma (U118MG) and osteosarcoma (HuO9, SAOS2), selecting cell lines based on LRRC15 gene expression data in publicly available databases: the EMBL-EBI expression atlas, Harmonizome 3.0, COSMIC, and the Cancer Cell Line Encyclopedia (CCLE) database.^11^ Despite selecting cell lines that exhibited high *LRRC15* expression at the RNA level, not all were found to have detectable protein on the cell surface. Among those that did, DUNP19 retained picomolar affinity for cell-surface target antigen (EC50 = 0.83–222.22 pM) (Fig. 1a). In addition, a positive correlation was noted between the number of LRRC15 molecules on the cell surface and *LRRC15* mRNA levels (R^2^ = 0.567) (Fig. 1b). Specific binding to LRRC15 on the cell surface was further confirmed by confocal microscopy of AF647-labeled DUNP19 (Fig. 1c). Next, we studied cellular internalization of DUNP19. Antibody internalization enhances the retention time of the delivered radionuclide; this can increase both image contrast and absorbed doses of therapeutic radiation.^12^ Confocal microscopy studies illustrated that DUNP19 is rapidly internalized by LRRC15 expressing cells (Fig. 1d, e). Interestingly, internalization rates for DUNP19 were contingent on the quantity of available molecules; faster kinetics were observed in cells with a higher abundance of LRRC15 molecules (HuO9: 1284.74 ± 63.17 molecules per µm^2^, 132.06 ± 10.14 min; SAOS2: 684.89 ± 100.67 molecules per µm^2^, 145.62 ± 15.18 min; Fig. 1d). Conjugation chemistry did not impact DUNP19 internalization; the internalization rate of the chelate-conjugated antibody (CHX-A”-DTPA-DUNP19) was analogous to that of the unconjugated antibody (Supplementary Fig. 2a). DUNP19 was also able to internalize into Hs819.T fibroblastic cells expressing *Lrrc15* (Supplementary Fig. 2b). Furthermore, time-resolved cellular assays indicated that ^177^Lu-radiolabeling did not affect DUNP19 binding to LRRC15 (U118MG: K_D_ = 301 ± 39 pM; HuO9: 117 ± 46 pM; SAOS2: 56 ± 27 pM; RPMI-7951: 22.1 ± 3.8 pM; (*n* = 2–3), Fig. 1f).

**Fig. 2:**
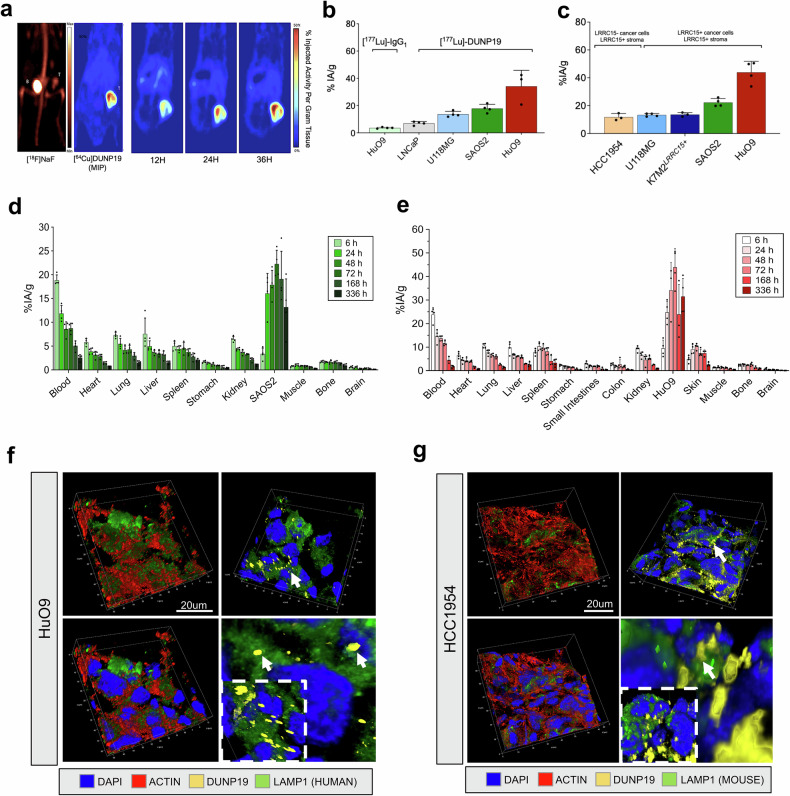
Specificity and biodistribution of DUNP19 in multiple mouse models with LRRC15 expressing tumors. **a** Representative PET images of s.c. SAOS2 osteosarcoma xenografts (*n* = 4) obtained at different time points post i.v. administration of [^64^Cu]Cu-DUNP19, highlighting high tumor uptake with minimal accumulation in normal tissues. In contrast, PET with the clinical bone scanning agent [^18^F]-NaF showed low activity in tumor tissue, with the majority of the radiotracer dose observed in bone (Bn) and bladder (Bl). **b** In vivo assessment of LRRC15 targeting specificity by [^177^Lu]Lu-DUNP19. At 48 h post i.v. injection, [^177^Lu]Lu-DUNP19 displayed significantly higher uptake (*p* = 0.001 and 0.005) in LRRC15+ U118MG (blue bar) and HuO9 (red bar) tumors compared to LRRC15- LNCaP tumors (light gray bar). The accumulation of non-specific [^177^Lu]-IgG1 in LRRC15+ HuO9 tumors (light blue bar) was significantly lower than that of [^177^Lu]Lu-DUNP19 (*p* = 0.003; *n* = 4/group). **c** [^177^Lu]Lu-DUNP19 tumor uptake in multiple s.c. tumor models at 72 h p.i., correlating with the LRRC15 expression level in the respective model. **d**, **e** Kinetics of [^177^Lu]Lu-DUNP19 in healthy organs and LRRC15+ SAOS2 and HuO9 osteosarcoma lesions (*n* = 4). Ex vivo tissue biodistributions of [^177^Lu]Lu-DUNP19 obtained at multiple time points after i.v. injection showed a continuous decline in activity levels in healthy organs, but sustained uptake by malignant lesions. **f**, **g** Microanatomy of tumor tissues obtained from animals treated with fluorescently labeled DUNP19. Confocal images of s.c. HuO9 (LRRC15+ cancer cells) and HCC1954 (LRRC15- cancer cells/LRRC15+ stroma) tumors harvested at 72 h post- i.v. injection of AF594-DUNP19 (yellow). Tumor sections were co-stained for Actin (red), DNA (DAPI, blue) and LAMP1 (lysosomal marker, green). Images show that DUNP19 accumulates in the cellular cytoplasm and co-localized with LAMP1 indicating intracellular trafficking of the mAb to the lysosomal compartments (arrow) after binding membranous LRRC15

### Tumor-associated LRRC15 expression by non-invasive PET

To evaluate in vivo kinetics of DUNP19 in healthy organs and LRRC15-expressing tumors, sequential PET images were acquired of subcutaneous (s.c.) osteosarcoma (SAOS2) bearing mice after intravenous (i.v.) administration of a ^64^Cu-labeled version of the mAb ([^64^Cu]Cu-DUNP19). Tumor accumulation was compared to the clinical bone scanning agent [^18^F]-NaF. Rapid [^64^Cu]Cu-DUNP19 uptake in vivo recapitulated in vitro internalization, selectivity, and retention in malignant tissue. In contrast, [^18^F]-NaF exhibited limited accumulation in osteogenic tumors, with the bladder showing the highest activity due to urinary excretion (Fig. 2a). Low accumulation of radioactivity in blood and organs known to sequester free ^64^Cu and ^177^Lu indicated stability of radiolabeled DUNP19 (Fig. 2 and Supplementary Fig. 3). These findings suggest that DUNP19-PET can be utilized to noninvasively determine LRRC15 expression and select patients for treatment, improving upon existing FDA-approved PET tracers for bone cancer lesions.

### [^177^Lu]Lu-DUNP19 exhibits a favorable biodistribution

First, the effect of antibody carrier mass on the biodistribution of [^177^Lu]Lu-DUNP19 in s.c. osteosarcoma (HuO9) tumors was evaluated. An injected antibody mass of 15–30 µg resulted in the most favorable tumor-to-normal tissue radioactivity uptake ratios. Next, we systematically examined the biodistribution and pharmacokinetic profile of [^177^Lu]Lu-DUNP19 in a variety of s.c. tumor models originating from diverse malignant tissues. These studies evaluated multiple cancer lineages with various levels of LRRC15 expression and with target expression in distinct tumoral compartments. Tumor models with LRRC15+ cancer cells included K7M2^*LRRC15+*^, HuO9 and SAOS2 osteosarcomas (OS) and U118MG glioblastoma (GBM). We also assessed [^177^Lu]Lu-DUNP19 uptake in the HCC1954 breast cancer model, characterized by high LRRC15 expression in stromal cells and LRRC15-null cancer cells (Fig. 2, Supplementary Fig. 4, Supplementary Tables 1, 2).

The accumulation of [^177^Lu]Lu-DUNP19 in tumors peaked at 72 h post-injection (p.i.) (HCC1954: 11.8 ± 2.5%IA/g [percent injected activity per gram tissue], U118MG: 13.3 ± 1.1%IA/g, K7M2^*LRRC15+*^: 13.6 ± 1.5%IA/g, SAOS2: 22.3 ± 2.9%IA/g, HuO9: 43.9 ± 7.9%IA/g) and remained consistently elevated at all studied time points up to 336 h p.i. (Fig. 2b, Supplementary Fig. 4). Retention of [^177^Lu]Lu-DUNP19 steadily decreased in blood and healthy organs after injection, and [^177^Lu]Lu-DUNP19 in blood reflected the expected half-life of a human IgG_1_ in mice, indicating interaction with the murine neonatal fragment crystallizable (Fc) receptor (FcRn).^13,14^ Retention of [^177^Lu]Lu-DUNP19 in the liver was representative of typical blood volume and metabolic elimination of antibodies. Taken together, these data indicate a favorable biodistribution profile of [^177^Lu]Lu-DUNP19 (Fig. 2d, e).

LRRC15 targeting specificity in vivo was further addressed in the s.c. LRRC15+ osteosarcoma tumor model HuO9. Uptake of [^177^Lu]Lu-DUNP19 was compared to non-specific [^177^Lu]-huIgG_1_, a human IgG_1_ with non-binding complementary-determining regions (CDRs) that had been radiolabeled with ^177^Lu. At 48 h post-i.v. administration, tumor uptake of [^177^Lu]Lu-DUNP19 was significantly higher than [^177^Lu]-huIgG_1_ (34.14 ± 11.72 vs. 3.62 ± 0.48%IA/g, respectively, *p* = 0.003) (Supplementary Fig. 5). Additionally, in LRRC15- s.c. LNCaP tumors, which lack relevant amounts of murine LRRC15 expressing stroma but are highly vascularized, systemic injection of [^177^Lu]Lu-DUNP19 resulted in tumor retention of 6.97 ± 1.3%IA/g at 48 h p.i. (Fig. 2b). These findings indicate that DUNP19 specifically targets LRRC15-expressing tumor tissue with minimal off-target retention in vivo. Accumulation of [^177^Lu]Lu-DUNP19 in LRRC15- LNCaP tumors is likely due to the enhanced permeability and retention effect. This pathophysiological mechanism involves the entrapment of macromolecules >45 kDa facilitated by the abnormally permeable and fenestrated-like tumor vasculature, resulting in a pronounced non-specific uptake of compounds in small animal xenograft tumor models as opposed to malignant lesions observed in humans.^14,15^

Next, we investigated the subcellular localization of DUNP19 in human osteosarcoma (SAOS2 and HuO9), and breast cancer (HCC1954) tumor models following systemic administration. Sections from s.c. tumors collected 72 h after i.v. injection of AlexaFluor647-labeled DUNP19 were co-stained for DNA, actin, and lysosomes (LAMP1) and analyzed by confocal microscopy (Fig. 2f, g, Supplementary Fig. 7). Consistent with our in vitro findings, DUNP19 co-localized with murine LAMP1 in HCC1954 tumors, and with human LAMP1 in HuO9 and SAOS2 tumors. This co-localization suggests cellular internalization of the antibody after binding with LRRC15 on the plasma membrane of both cancer and stromal cells.

### [^177^Lu]Lu-DUNP19 radioimmunotherapy (RIT) of aggressive osteosarcoma

**Fig. 3:**
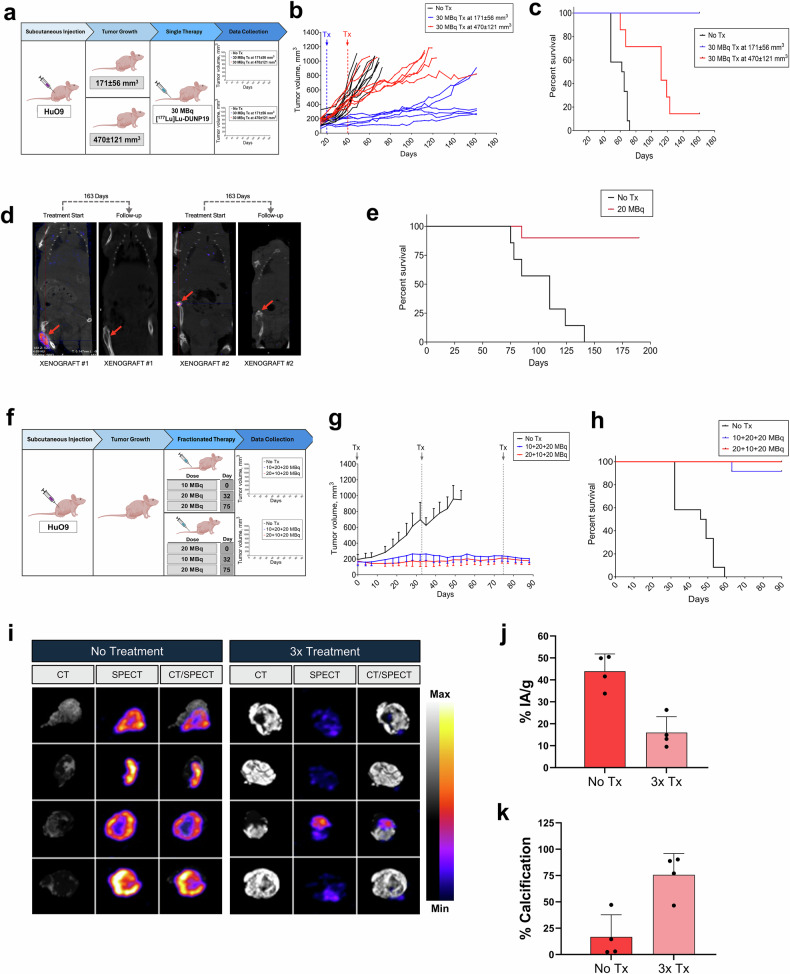
Evaluation of [^177^Lu]Lu-DUNP19 therapy and monitoring in HuO9 osteosarcoma tumors. **a**–**c** Schematic illustration (created with BioRender.com) of the experimental design and results (**a**). Tumor volumes in mice bearing s.c. HuO9 xenografts were randomized for a single i.v. administration of 30 MBq [^177^Lu]Lu-DUNP19 when tumors reached 171 ± 56 mm^3^ (blue line; day 19, *n* = 6) or 470 ± 121 mm^3^ (red line; day 40, *n* = 7) or received no treatment (black line). The results demonstrated a significant delay in disease progression, with treatment efficacy being influenced by tumor volume (*p* *<* 0.0001; group 1 vs. group 2) (**b**). Kaplan–Meier survival analysis revealed that [^177^Lu]Lu-DUNP19 extended survival, with the impact varying based on the timing of intervention (**c**). **d**, **e** Representative maximum intensity projection coronal SPECT/CT images showing orthotopic HuO9 osteosarcoma tumors (indicated by arrow) after initial (left) and follow-up (right) i.v. administration of 20 MBq [^177^Lu]Lu-DUNP19. In all treated mice (right) (*n* = 10), no tumor associated uptake was observed at 163 days after treatment (vehicle *n* = 7) (**d**). Kaplan–Meier survival analysis revealed a significant increase in survival for the [^177^Lu]Lu-DUNP19 treated group during the observed 190-days period (*p* = 0.0002) (**e**). **f**–**k** Illustration of the experimental setup and key findings (created with BioRender.com) (**f**). Mice with s.c. HuO9 osteosarcoma xenografts (*n* = 12 per arm) were randomized for three treatment cycles (blue: 10 + 20 + 20 MBq; red: 20 + 10 + 20 MBq) of i.v. [^177^Lu]Lu-DUNP19 (at day 0, 32, and 75), resulting in a total administered activity of 50 MBq, or no treatment (control; black). Assessment of tumor volumes demonstrated that repeated cycles of LRRC15-RIT effectively inhibit tumor growth (**g**). Kaplan–Meier survival analysis confirmed significantly improved survival for mice randomized for [^177^Lu]Lu-DUNP19 over no treatment (*p* < 0.0001) (**h**). Four tumors from the treatment and untreated controls, harvested 72 h after administration of an imaging dose of [^177^Lu]Lu-DUNP19 (3.5 MBq), were imaged ex vivo by SPECT and CT (**i**). Tissue activity levels (%IA/g), assessed by gamma counter and normalized to tissue weight, revealed significantly lower uptake of the [^177^Lu]Lu-DUNP19 in treated vs. non-treated tumors (*p* = 0.002) (**j**). Quantification of radiopacity in CT images showed significantly higher ossification levels in treated vs. non-treated tumor tissues (*p* < 0.01) (**k**). Together, these findings illustrate that repeated cycles of [^177^Lu]Lu-DUNP19 effectively reduce tissue viability and increase calcification

We then investigated the impact of [^177^Lu]Lu-DUNP19 radioimmunotherapy (RIT) on tumor volume and overall survival in mice bearing HuO9 osteosarcoma tumors. Following a single systemic administration of 30 MBq [^177^Lu]Lu-DUNP19, tumor growth was significantly inhibited in mice with an initial tumor volume of 171 ± 56 mm³, resulting in prolonged survival (*p* *<* 0.0001, Fig. 3b). Infiltrative and bulky osteosarcoma have poorer prognoses and limited treatment options. To challenge our RIT a second cohort of animals bearing larger tumors (470 ± 121 mm³) received the same treatment regimen. In this group, disease progression was again delayed relative to controls, and overall survival improved to a median of 112 days (range: 60–161 days, *p* = 0.003). All untreated animals succumbed by 72 days (range: 48–72 days) (Fig. 3c).

We evaluated the impact of sequential and fractionated dosing on tumor growth and survival. From a translational perspective, this approach is commonly utilized in clinical settings to optimize maximum tolerated dose while reducing dose-limiting toxicities.^16–18^ Fractionation is also recommended to compensate for the anticipated heterogeneity in RIT dose distribution, particularly in large poorly vascularized tumors with regions of hypoxia.^19^ Rather than adhering to a predetermined activity and treatment schedule, additional therapeutic doses were given based on recovery from bone marrow toxicity determined by measuring differential blood counts, regrowth of tumor volume, and their effect on animal overall weight (Supplementary Figs. 8, 9). Mice bearing subcutaneous HuO9 tumors (169 ± 44 mm^3^) were administered a cumulative activity of 50 MBq in three fractions over a span of 75 days (Fig. 3g). Continuous progression-free survival was sustained in treated animals throughout the study duration, in contrast to untreated animals which exhibited a median survival of 47.5 days (*p* < 0.0001; Fig. 3h).

We further studied LRRC15-targeted RIT in a translationally relevant orthotopic osteosarcoma model (Fig. 3d, e). HuO9 cells were injected into the left tibia of Balb/c mice and subjects were randomly selected for systemic injection with 20 MBq of [^177^Lu]Lu-DUNP19 23 days after inoculation. Imaging by SPECT at 72 h post-[^177^Lu]Lu-DUNP19 administration revealed LRRC15-specific accumulation of activity at the tumor site (Fig. 3d, e). Nine of ten treated animals survived to the study endpoint (190 days), while all untreated animals succumbed due to disease-related endpoints (*p* = 0.0002; Fig. 3e). Follow-up imaging with [^177^Lu]Lu-DUNP19 was acquired 163 days after treatment and revealed no accumulation of the theranostic agent at the site of HuO9 tumor inoculation or at other anatomical locations (Fig. 3d). Radiation is widely known to induce calcifications in sarcomatous processes.^20^ To investigate this phenomenon in animals bearing HuO9 lesions, we quantified tumor radiopacity and uptake of tumor-associated [^177^Lu]Lu-DUNP19 using SPECT/CT and gamma-spectrometry. Animals treated with [^177^Lu]Lu-DUNP19 and untreated mice received a subtherapeutic imaging dose of 3.5 MBq [^177^Lu]Lu-DUNP19 and tumors were harvested 72 h after injection. Treated tumors exhibited significantly lower tumor-associated [^177^Lu]Lu-DUNP19 activity (*p* = 0.002), coupled with higher levels of tumor calcification (Fig. 3i–k).

### [^177^Lu]Lu-DUNP19 therapy is effective across a range of tumors with varying LRRC15 expression patterns

**Fig. 4:**
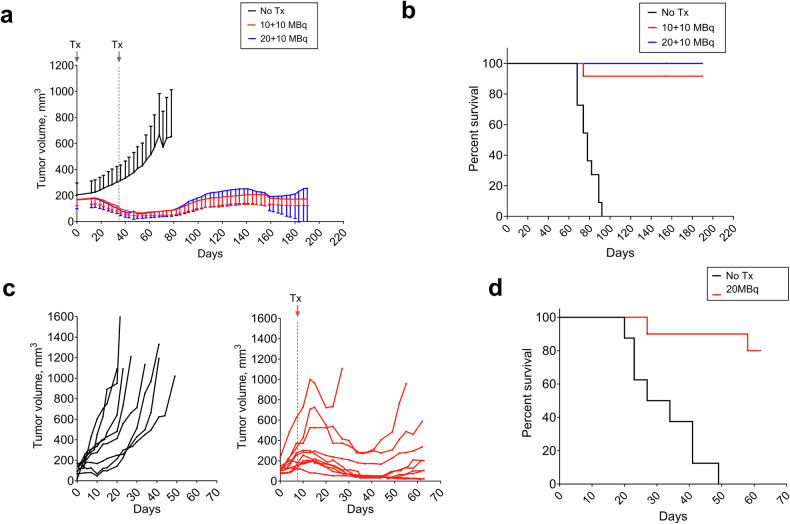
Therapeutic efficacy of [^177^Lu]Lu-DUNP19 in LRRC15-expressing human xenograft models. **a**, **b** BALB/c nude mice bearing s.c. U118MG GBM xenografts were treated with two fractions of [^177^Lu]Lu-DUNP19 at days 0 and 34 for a cumulative activity of 20 MBq (10 + 10 MBq, red, *n* = 12) or 30 MBq (20 + 10 MBq, blue, *n* = 12) or left untreated (*n* = 11). Despite lower LRRC15 expression by U118MG tumors, treatment with [^177^Lu]Lu-DUNP19 significantly controlled tumor growth and prolonged survival (median survival; untreated = 78 days, 20 MBq = not reached, 30 MBq = not reached, *p* < 0.0001). **c** [^177^Lu]Lu-DUNP19 is effective in HCC1954 breast cancer models (LRRC15- cancer cells, LRRC15+ stroma). Results demonstrate delayed s.c. HCC1954 growth in female mice intravenously administered a single dose of [^177^Lu]Lu-DUNP19 (20 MBq; day 8, *n* = 10). **d** Median survival was not reached for the treated group by the end of the observation period (day 62), while median survival of control mice was 30.5 days (*p* = 0.0001)

Given the high expression of LRRC15 on HuO9 osteosarcoma cells (Fig. 1, Supplementary Fig. 6), we also sought to understand how the effects of [^177^Lu]Lu-DUNP19 therapy would change in a tumor of different tissue origin and with lower LRRC15 expression. Mice bearing s.c. U118MG GBM-xenografts were treated with a cumulative activity of 20 MBq or 30 MBq [^177^Lu]Lu-DUNP19 in two fractions. [^177^Lu]Lu-DUNP19 treatment significantly extended survival (*p* < 0.0001), and therapy prevented further tumor growth as tumor volumes reached a plateau phase around 100–200 mm^3^. All untreated mice were euthanized due to disease-related endpoints by day 78, whereas only one mouse in the 20 MBq [^177^Lu]Lu-DUNP19 treatment group was euthanized, and all mice in the 30 MBq group survived until the end of the observation period (Fig. 4a, b). Finally, we investigated the therapeutic efficacy of [^177^Lu]Lu-DUNP19 in the HCC1954 breast cancer model, composed of LRRC15- cancer cells supported by LRRC15+ stroma. Despite LRRC15 expression in stroma only, a single systemic injection of 20 MBq [^177^Lu]Lu-DUNP19 effectively suppressed HCC1954 tumor growth and significantly prolonged median survival compared to untreated mice (*p* = 0.0001; median survival not reached vs. 30.5 days). Notably, 80% of the treated mice (8/10) survived until the end of the study (Fig. 4c, d). Given the lack of target expressing cancer cells, the response of HCC1954 tumors to [^177^Lu]Lu-DUNP19 is reliant on targeting of LRRC15+ murine CAFs. Throughout all studies, treatment was generally well-tolerated as indicated by stable average body weights (Supplementary Fig. 8). Administration of [^177^Lu]Lu-DUNP19 resulted in a transient bone marrow suppression, which recovered to baseline levels within 3–4 weeks (Supplementary Fig. 9).

### LRRC15-targeted RIT depletes TGFβ-driven signature in tumors

**Fig. 5:**
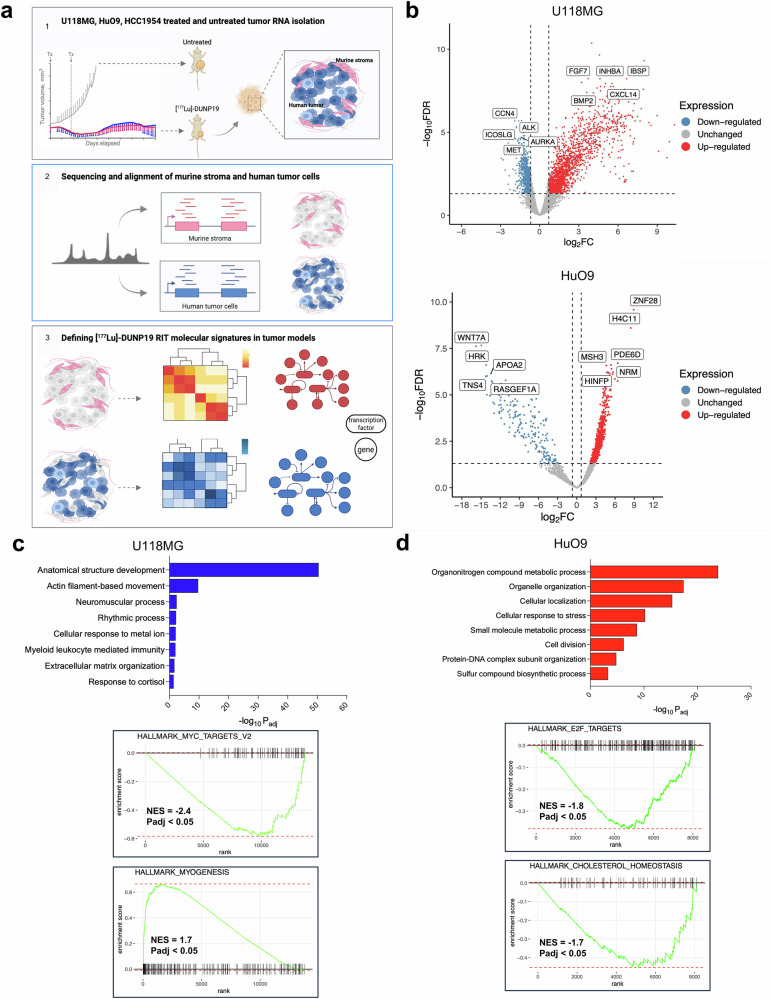
[^177^Lu]Lu-DUNP19 RIT induced signatures in LRRC15+ cancer cells. **a** Schematic of transcriptomic analysis of HuO9, U118MG, and HCC1954 tumors after [^177^Lu]Lu-DUNP19 treatment (HuO9, untreated *n* = 6, treated *n* = 16; U118MG, untreated *n* = 8, treated *n* = 16; and HCC1954, untreated *n* = 8, treated *n* = 10). Treated or untreated tumor samples were harvested for RNA isolation, before sequencing and alignment to either murine or human genomes. Overlapping or ambiguous reads were discarded. **b** Volcano plot of the top up- (red) and downregulated (blue) DEGs after treatment with [^177^Lu]Lu-DUNP19 in U118MG (left; *n* = 16) and HuO9 (right; *n* = 16) cancer cells (FDR < 0.05). DEGs were ranked by fold-change. The top and bottom genes were labeled. **c**, **d** Gene ontology (GO) biological pathway enrichment analysis of DEGs in treated (**c**) U118MG and (**d**) HuO9 cancer cells (adjusted *p*-value < 0.05). Enriched biological pathways with more than 10 overlapping terms (genes) were plotted by adjusted *p*-value value to indicate processes most significantly enriched after [177Lu]Lu-DUNP19 RIT

Having demonstrated the significant therapeutic potential of our radioimmunotheranostic platform, we sought to understand the molecular effects of LRRC15-targeted RIT on cancer cells and the tumor microenvironment. We harvested tumors at 62–116 days (HuO9, HCC1954) or 155–190 days (U118MG) after [^177^Lu]Lu-DUNP19 treatment, i.e., at a time when tumor volumes had plateaued and no additional tumor growth was observed, to gain insight into the transcriptome of the remaining tumor tissue, which often contributes to resistance and recurrence in patients. Transcripts of bulk RNA-sequencing were aligned to human and murine genomes to identify the transcriptomic profiles of human cancer cells and murine stromal cells. Ambiguous reads were subsequently removed (Fig. 5a). U118MG tumors were also harvested at 30 days post-RIT treatment (at tumor volume nadir) to enable us to investigate the earlier effects of LRRC15-RIT, while the later time point provided insight into the transcriptome of the remaining tumor tissue, which often contributes to resistance and recurrence in patient settings. Due to the fractionated dosing regimens utilized in our therapeutic studies (Figs. 3, 4), our first objective was to determine whether there were significant differences in gene signatures that resulted from the different [^177^Lu]Lu-DUNP19 dosing regimens. We did not find gene signatures that were associated with the absorbed dose [^177^Lu]Lu-DUNP19. Therefore, in subsequent analyses and for each model, [^177^Lu]Lu-DUNP19-treated tumors were compared to untreated tumors.

In human cancer cells, RNA-sequencing analysis identified 19,578 protein-coding genes, of which 1985 (HuO9), 1043 (U118MG), and 23 (HCC1954) were differentially expressed genes (DEGs) after [^177^Lu]Lu-DUNP19 therapy. In [^177^Lu]Lu-DUNP19-treated murine stroma, 663 (HuO9), 319 (U118MG), and 73 (HCC1954) DEGs were identified compared to untreated controls (Fig. 5b). In U118MG tumors, the most upregulated genes included bone morphogenic protein (*BMP2*) and inhibin subunit beta A (*INHBA*), two modulators of TGFβ signaling and epithelial-mesenchymal transition (Fig. 5b) (FDR < 0.05). Treatment with [^177^Lu]Lu-DUNP19 RIT in HuO9 tumors resulted in increased expression of epigenetic regulators, including HINFP and H4C11. In contrast, the expression of anti-apoptotic genes, such as HRK, and genes within the WNT and RAS signaling pathways, which are associated with tumor promotion, was substantially reduced (Fig. 5b) (FDR < 0.05).

To gain a sense of the biological pathways altered by [^177^Lu]Lu-DUNP19 RIT, gene ontology analysis of DEGs from tumors containing LRRC15+ cancer cells (U118MG, HuO9) was performed (Fig. 5c, d). In treated U118MG tumors, gene ontology terms were immune- and epithelial-mesenchymal transition related (response to cortisol, myeloid leukocyte mediated immunity, anatomical structural development) (Fig. 5c). Further analysis using gene set enrichment analysis (GSEA) revealed enrichment of myogenesis (myoblast stem cell differentiation) and a downregulation of MYC target genes (Fig. 5c, Supplementary Table 3). In contrast, GSEA and gene ontology analysis of treated HuO9 tumors revealed alterations in metabolic and cell cycle-related pathways, as indicated by negative enrichment of E2F cell cycle transcription factors, as well as alterations in nitrate metabolism, lipoprotein homeostasis, and response to stress (Fig. 5d, Supplementary Table 3). Despite differences in the biological pathways involved in [^177^Lu]Lu-DUNP19 RIT response, 40 genes were differentially expressed in both U118MG and HuO9 treated tumors (Supplementary Fig. 14). Of the 40 shared genes, several had functions similar to the hypothesized roles of LRRC15 (cell migration, invasion, and adhesion) or had been shown to be co-expressed with *LRRC15*, including *COL11A1*, *FGF13*, and *CXCL14* (Supplementary Fig. 14).^8,21^

**Fig. 6:**
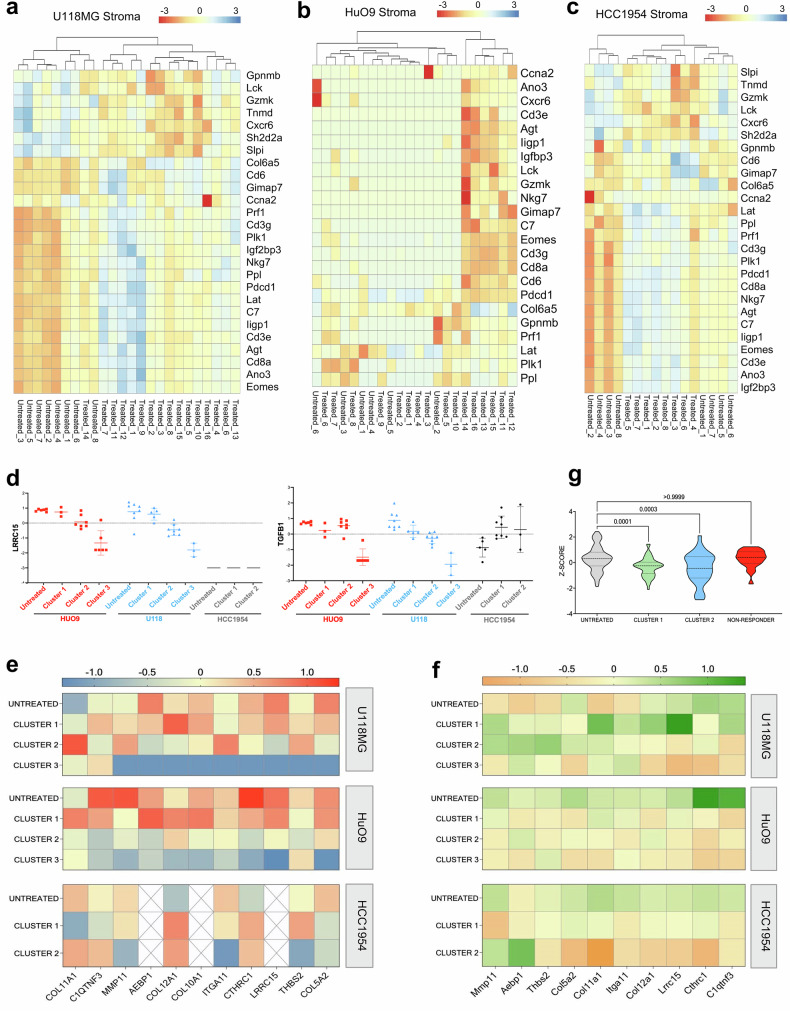
[^177^Lu]Lu-DUNP19 therapy decreases expression of a LRRC15 + TGFβ signature associated with immunotherapy resistance. **a**–**c** DEGs overlapped in [^177^Lu]Lu-DUNP19-treated stroma from U118MG (**a**, 26 genes; *n* = 16 tumors), HuO9 (**b**, 23 genes; *n* = 16 tumors), and HCC1954 (**c**, 26 genes; *n* = 10 tumors) tumors. Relative expression (Z-score normalization) was plotted to indicate upregulated (red) or downregulated (blue) genes. **d** Box-and-whisker plots representing relative transcript expression of LRRC15 (top) and TGFB1 (bottom), comparing untreated tumors to tumors after [^177^Lu]Lu-DUNP19 therapy. HuO9 transcripts are plotted in red (left), U118MG in blue (middle), and HCC1954 in black (right). Samples were separated by transcript signature based on PCA plots and hierarchical clustering (Supplementary Fig. 10) into 2 (HCC1954) or 3 (U118MG, HuO9) clusters. Expression of LRRC15 and TGFβ1 in treated samples from cluster 3 are significantly (*p* < 0.005) decreased in U118MG and HuO9, while no changes are observed in the LRRC15- HCC1954 cancer cells. **e**, **f** Transcript data from clustered (Supplementary Fig. 10) cancer cells (**e**) or tumor stroma (**f**) show decreased expression of the LRRC15+ TGFβ signature. **e** Untreated U118MG (top) and HuO9 (middle) cancer cells lose expression of the LRRC15 + TGFβ signature after [^177^Lu]Lu-DUNP19 treatment (red = high, blue = low expression). **f** Loss of the LRRC15+ TGFβ signature is observed across all tumor stroma after [^177^Lu]Lu-DUNP19 RIT (green = high, orange = low expression). **g** HCC1954 tumors that were resistant to [^177^Lu]Lu-DUNP19 treatment (defined as reaching 1000 mm^3^ endpoint before conclusion of study, *n* = 2) had no significant reduction of the 11-gene LRRC15+ TGFβ signature within tumor stroma (*p* < 0.05)

To complement our understanding of transcriptomic responses to [^177^Lu]Lu-DUNP19 RIT in the tumor microenvironment, we also conducted a comprehensive analysis of the murine stroma in HuO9, U118MG, and HCC1954 tumors. In all three models, RIT induced changes in pathways related to immune activation, including the upregulation of *Gzmk*, *Cxcr6*, and *Lck* (Fig. 6a–c). Whole transcriptome principal component analysis identified three distinct transcriptional clusters for treated cancer cells in the HuO9 and U118MG models and two clusters in HCC1954 (Supplementary Fig. 10). Based on these results, we studied whether these changes could be explained by an overall shift in cell types present within the [^177^Lu]Lu-DUNP19 treated tumor samples (i.e., loss of mesenchymal phenotypes). We employed Syllogist to further assess the proportional distribution of cell types in treated tumors compared to untreated samples.^22^ In accordance with the expression of *LRRC15* in cancer cells originating from mesenchymal stem cells, HuO9 and U118MG tumors displayed a notable overrepresentation of mesenchymal cells across all examined samples, with a significant loss of the mesenchymal cell phenotype in [^177^Lu]Lu-DUNP19-treated HuO9 cancer cells (Supplementary Fig. 12). No discernible alterations in relative cell composition were observed following treatment with [^177^Lu]Lu-DUNP19 in other tumor models. Additionally, our observations revealed that HCC1954 tumor cells predominantly maintained an epithelial phenotype, consistent with the absence of *LRRC15* expression in the cancer cells (Supplementary Fig. 11).

Overall, and in line with LRRC15 protein levels (Fig. 1b, c; Supplementary Fig. 6), *LRRC15* expression was higher in HuO9 than in U118MG cancer cells, and not quantifiable in HCC1954 cancer cells. In contrast, stromal *Lrrc15* expression was 3- and 4-fold higher in HCC1954 tumors than in U118MG and HUO9 tumors, respectively (Supplementary Fig. 12). Comparison of *LRRC15/Lrrc15* expression in cancer cells and stroma of HuO9 and U118MG tumors across clusters showed a trend of decreased expression with increasing cluster distance from untreated samples in both cancer cells and stroma (Fig. 6d); the expression of *TGFB1*, a known regulator of LRRC15 expression, mirrored the *LRRC15/Lrrc15* expression pattern (Fig. 6d). Interestingly, *Lrrc15* and *Tgfb1* levels in HCC1954 stroma remained constant, while *TGFB1* expression was increased in treated LRRC15-null HCC1954 cancer cells.

Further analysis of LRRC15+ clusters showed that treatment with [^177^Lu]Lu-DUNP19 resulted in the progressive loss of expression of a gene signature associated with immune cell exclusion and poor response to immune checkpoint blockade in TGFβ-driven, LRRC15+ CAFs^4,8^ (Fig. 6e, f). Consistently, in two mice with HCC1954 tumors which progressed despite [^177^Lu]Lu-DUNP19 RIT, neither *Lrrc15* nor the LRRC15 + CAF gene-signature decreased (Fig. 6h).

Finally, to capture earlier treatment effects, bulk RNA-seq data from U118MG tumors treated with a single injection of RIT and harvest at nadir (30 days p.i.) were collected. The expression of several LRRC15-related genes known to contribute to immune-checkpoint inhibitor resistance (e.g., COL10A1, COL11A1, COL12A1, MMP11) was higher than in tumors harvested 155–190 days post RIT administration, suggesting a long-term benefit from [^177^Lu]Lu-DUNP19 (Supplementary Fig. 13). LRRC15 expression was already reduced at this early time point, indicating that its downregulation occurs rapidly following treatment. A major challenge with immune checkpoint inhibitors are tumors that harbor an “immune-excluded” microenvironment, characterized by CD8 + T cells that have accumulated but not efficiently infiltrated into the center, in large part due to the presence of reactive stroma driven by TGFβ signaling.^23^ Together, these findings suggest that targeting LRRC15 with [^177^Lu]Lu-DUNP19 could remodel the tumor microenvironment, potentially creating an opportunity to combine this radiotherapeutic agent with immune checkpoint inhibitors.

**Fig. 7:**
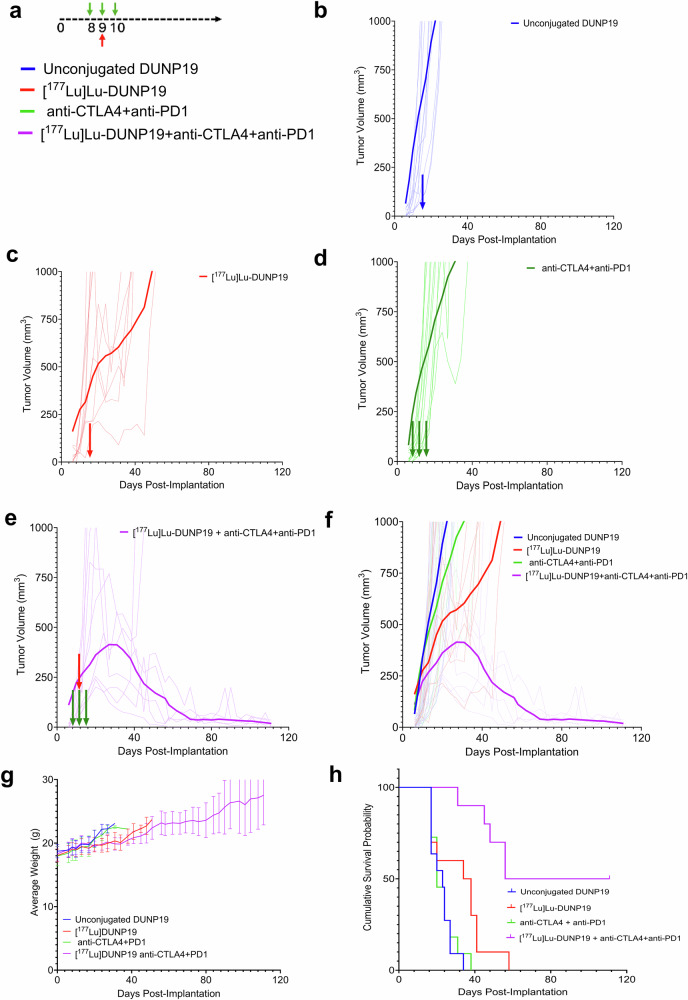
Combination of [^177^Lu]Lu-DUNP19 therapy and immune checkpoint blockade slows tumor growth and prolongs survival in a syngeneic colorectal adenocarcinoma mouse model. **a** Schematic of combination therapy study. **b** Tumor growth for murine colorectal adenocarcinoma (MC38) bearing mice (*n* = 8 mice/group) following treatment with unlabeled DUNP19 (blue, control), **c** [^177^Lu]Lu-DUNP19 RIT (7.4 MBq), **d** anti-CTLA4 and anti-PD1 treatment (100 μg, on three consecutive days), and **e** combined RIT and anti-CTLA4 and anti-PD1 therapy. **f** The moving average of the groups is plotted over the course of the experiment illustrating separation of treatment groups. **g** All groups tolerated the treatments well and no loss of body mass was reported in any group. No subjects were censored for lethargy or weight loss throughout the study. **h** Cumulative survival plotted over the course of the study by treatment group

To investigate whether [^177^Lu]Lu-DUNP19 therapy could synergize with immune checkpoint inhibitors, we combined [^177^Lu]Lu-DUNP19 RIT with immune checkpoint blockade (anti-CTLA4 + anti-PD1) in the syngeneic MC38 colorectal cancer model (LRRC15- cancer cells, LRRC15+ stroma) (Fig. 7). In agreement with the transcriptomic changes, RIT induced tumor growth delay and prolonged survival (36 days vs. 23 days in the control group, *p* < 0.01), and synergized with anti-CTLA4 and anti-PD-1 therapy (median survival >65 days, *p* < 0.001, vs. RIT alone).

## Discussion

LRRC15 has emerged as a promising TGFβ-driven biomarker expressed on the cell membrane of cancer cells derived from mesenchymal stem cells and on a subset of CAFs within the tumor microenvironment.^4,8^ Studies evaluating genes associated with metastatic progression have characterized LRRC15’s role in metastasis to bone in breast cancer and to bowel in ovarian cancer, while LRRC15 knockdown by siRNA significantly inhibited tumor progression in preclinical models.^24^ Furthermore, a retrospective study assessing primary osteosarcoma lesions identified a correlation between LRRC15 expression, aggressive disease, and shorter overall survival.^9^

The novel technology presented here harnesses the unique characteristics of a humanized IgG_1_ antibody, DUNP19, to specifically bind to a phylogenetically stable epitope of LRRC15. DUNP19’s picomolar affinity for human LRRC15 aligns with that of clinically successful antibodies and ensures robust binding to human LRRC15, as demonstrated in vitro and in xenograft models. While DUNP19 has a weaker affinity for murine LRRC15, our in vivo data reveal efficient targeting of murine LRRC15 + CAF and human LRRC15+ xenografts, indicating that avidity effects mitigate this limitation (see Supplementary Materials for a detailed discussion of DUNP19’s affinity). The rapid cellular internalization of DUNP19 by LRRC15-expressing cells provides an optimal foundation for leveraging DUNP19 as an effective vehicle for delivery of radionuclides to target cells. In addition, the IgG1 format employed here offers several compelling advantages such as antibody-dependent cellular cytotoxicity (ADCC) and complement-dependent cytotoxicity (CDC), which can be further amplified in combination with radiotherapy. Beyond these considerations, the DUNP19 mAb platform readily supports adaptation into other modalities, such as antibody-drug conjugates (ADCs) and enhanced ADCC-based therapies, leveraging the same high-affinity LRRC15-targeting framework (see Supplementary Materials for a detailed discussion of the capabilities of the IgG1-based mAb platform).

Our LRRC15-RIT approach effectively targeted various cancer models, including models of breast cancer, osteosarcoma, glioblastoma, and colorectal cancer, all representing highly lethal cancers with distinct tumor biology and LRRC15 expression patterns. Notably, the specific depletion of LRRC15+ cells through a single systemic administration of [^177^Lu]Lu-DUNP19 significantly slowed tumor progression and conferred a survival benefit in all models. These outcomes align with previous observations where the genetic ablation of LRRC15+ CAFs in murine models of pancreatic adenocarcinoma reduced tumor volume and slowed tumor growth.^8^ Moreover, in vivo cellular internalization has been reported to enhance radioisotope retention and reduce extracellular dissociation of the radioconjugate.^25^ In agreement with other beta-emitting RIT, we noted a decrease in white and red blood cells after administration of [^177^Lu]Lu-DUNP19.^26^ However, the bone marrow recovered within an expected time frame after treatment injection and body weights remained stable throughout the studies, allowing for serial dosing.

The LRRC15 protein is representative of a distinct set of TGFβ-driven genes that are predictors of immune checkpoint blockade resistance and unfavorable tumor evolution.^4,8^ Given LRRC15 known association with TGFβ, immunosuppression, and the observed potential immunomodulatory effects of RIT,^27^ our second aim was to explore whether the anti-tumor activity induced by [^177^Lu]Lu-DUNP19 could reverse the signaling profile associated with immunosuppression and resistance to immunotherapy. To target tumor-supporting immunosuppressive stroma, many investigations have focused on fibroblast activation protein-α (FAP), a membrane bound serine protease overexpressed by CAFs.^28^ While both LRRC15 and FAP have been shown to be upregulated by TGFβ,^29^ recent studies revealed a unique subset of TGFβ-driven CAFs that express LRRC15,^4,8^ and that these LRRC15+ CAFs are associated with CD8 + T-cell exclusion.^8^ Our therapeutic studies, a notable observation emerged: LRRC15+ tumors exhibited a concurrent reduction in both growth and the TGFβ–LRRC15 gene signature upon exposure to RIT. However, within a small subset (2 out of 12) of breast cancer (HCC1954) cases, tumor volumes did not decrease following the administration of [^177^Lu]Lu-DUNP19. Moreover, this subset exhibited a persistent TGFβ–LRRC15 signature, suggesting a nuanced response within this specific context; further studies into the mechanisms behind the TGFβ–LRRC15 pathway and LRRC15 as a biomarker for TGFβ signaling are ongoing.^30^

We also show that LRRC15-targeted RIT leads to differential expression of genes in stromal cells related to immune cell function, including genes indicative of activation and proliferation of T-cells (*Cd3, Cd8, Gimap7, Gzmk, Lat, Lck, Eomes*) and natural killer cells (*Cxcr6, Eomes, Prf1*). In the stroma of U118MG tumors, the T-cell suppressor gene encoding PD-1 was downregulated, suggesting that [^177^Lu]Lu-DUNP19 may contribute to relief of immune cell suppression and T-cell exclusion, and that immune-based adjuvant therapies may be complementary to LRRC15-RIT. The transcriptomic remodeling of immune-related signaling pathways and expression of immune activating cytokines after RIT may increase immune cell activation and infiltration in previously immunologically “cold” tumors. Additionally, ablation of radio-resistant stroma may further increase immune cell invasion and help amplify the anti-tumor immune response. These data are in line with a recent study that investigated a theranostic approach built on a radioligand binding to FAP in tumor mouse models derived from the subcutaneous co-injection of murine tumor cells and immortalized fibroblast cell lines overexpressing human FAP.^31^ In this investigation, radiopharmaceutical therapy resulted in transiently increased PD-L1 levels on tumor cells and improved anti-tumor efficacy of the radioligand when combined with anti-PD-L1 immune checkpoint therapy. Mechanistic studies revealed modulation of the tumor microenvironment to a more immune activated state by combination therapy. In agreement with this, we show efficacy of our LRRC15-targeted radioimmunotheranostic platform in mouse models with LRRC15+ cancer cells (HuO9, U118MG) and with LRRC15+ murine stroma (CAF; HCC1954), leading to treatment-induced reversal of pro-tumorigenic mechanisms, modulation of immune-related pathways, and enhanced response to immune checkpoint inhibitors (ICIs).

In the broader context of cancer treatment, our findings provide a novel technique for non-invasive imaging and treatment of a wide range of aggressive tumors with limited options for targeted therapy. Critically, we have demonstrated that [^177^Lu]Lu-DUNP19 therapy can be effective despite heterogenous LRRC15 expression within tumor tissue, and in contexts where the tumor cells themselves do not express the target. The principle of crossfire from the beta particle emitting radioimmunotherapy within tumor tissue induces robust antitumor responses and enhances sensitivity to immune checkpoint blockade.

In addition, LRRC15’s regulation by TGFβ allows for successful targeting of pro-tumorigenic TGFβ signaling mechanisms that have been shown to contribute to immunotherapy resistance and poor prognosis. Given the safety challenges that historically have been encountered with direct targeting of TGFβ1 in clinical trials (e.g., bleeding and cardiovascular toxicity),^32^ targeting TGFβ-driven fibrosis via LRRC15-targeted RIT represents an attractive potential alternative. We show, at a transcriptomic level, that these TGFβ-LRRC15 signatures are largely erased in [^177^Lu]Lu-DUNP19-treated tumors and expression of other anti-tumor immune pathways increases. Based on these observations, we hypothesized that the eradication of the TGFβ-LRRC15 signature in tumor cells would be synergistic with existing immunotherapies and allow for immune cell infiltration, similar to observations by Krishnamurty et al.^8^ The functional effects of the loss of this transcriptomic signature were investigated by our combination study of [^177^Lu]Lu-DUNP19 and immune checkpoint blockade. This combination prolonged survival in a syngeneic tumor model, suggesting a synergistic effect between the two therapies. However, further studies are needed to explore how [^177^Lu]Lu-DUNP19-RIT promotes immune cell infiltrate and induces transcriptomic changes.

In summary, we propose targeting LRRC15+ cells with [^177^Lu]Lu-DUNP19 as a novel theranostic strategy that provides sustained tumor control across models of LRRC15+ disease, improves survival, and reprograms the transcriptomic landscape of pro-tumorigenic and immunosuppressive mechanisms within the TME, all with minimal side effects.

## Materials and methods

### Reagents

All reagents were purchased from Sigma-Aldrich unless otherwise stated.

### DUNP19

For DUNP19 production, HEK293 cells were cultured in a 2 L suspension using FreeStyle 293 Expression Medium (Life Technologies, Carlsbad, CA, USA) with a cell density maintained at 1 × 10^6^ cells/mL on the day of transfection. Expression plasmids harboring genes for DUNP19 heavy chain and light chain in human IgG1/kappa format were combined with the transfection agent and incubated for 10 min at room temperature. The transfected cell culture was then incubated at 37 °C, 8% CO_2_ on an orbital shaker rotating at approximately 110 rpm for seven days. Culture medium was collected, subjected to centrifugation, and filtered through 0.22 µm filter systems. Antibodies were isolated through Protein A chromatography, followed by buffer exchange to PBS (pH 7.4) via gel filtration. The concentration was calculated from absorbance measurement at 280 nm.

For labeling with Lutetium-177 and Copper-64, respectively, DUNP19 was functionalized with benzylisothiocyanate derivatives of the acyclic chelating agent CHX-A”-DTPA (p-SCN-CHX-A”-DTPA) or macrocyclic NOTA (p-SCN-NOTA), respectively, using amine-reactive chemistry. Radiolabeling was performed as previously described (see Supplementary Materials).^33^ The average radiochemical yield was 95 ± 5% with a radiochemical purity >99% and a maximum specific activity of 4 MBq/µg. Injection solutions of [^177^Lu]Lu-DUNP19 were formulated in 2% BSA/PBS containing 0.14 mM Na_2_EDTA (100-fold molar excess). Binding affinity of the radio-conjugate to life cells was determined using Ligandtracer technology.^34^ To evaluate the stability of the therapeutic agent [¹⁷⁷Lu]Lu-DUNP19, we analyzed the percentage of radiometabolites in blood samples collected 72 h after antibody administration. Plasma was separated from whole blood by centrifugation at 30,000 rpm for 10–15 min at 4 °C. The plasma was then processed using NAP-5 size-exclusion columns (5 kDa molecular weight cutoff), pre-equilibrated with 1% albumin in PBS, to separate high molecular weight (HMW, >5 kDa) components from low molecular weight (LMW, <5 kDa) components.

An amine-based protein labeling kit (Invitrogen, #A20173) was used for labeling DUNP19 with AlexaFluor-647 or AlexaFluor-594. Labeling was performed as described by the kit’s recommended protocol.

### Cell culture

U118MG (glioblastoma), U87MG (glioblastoma), RPMI7951 (melanoma), NCI-H196 (small cell lung cancer), HCC1954 (breast cancer), SAOS2 (osteosarcoma), U2OS (OS), Kasumi-2 (leukemia), Calu-1 (non-small-cell lung cancer), RCH-ACV (leukemia), MHH-Call-3 (leukemia), Hs737.T (giant cell sarcoma), HEK293T, LNCaP (prostate cancer), K7M2 (murine osteosarcoma) and MC38-luc (murine colorectal cancer) were purchased from ATCC. HuO9 (osteosarcoma) was purchased from the Japanese Cancer Research Resources Bank (Tokyo, Japan). All cell lines were cultured according to the manufacturer’s instructions and frequently tested for Mycoplasma.

To overexpress LRRC15, HEK293T and K7M2 cells were transduced with a pLenti-LRRC15-GFP-Puro vector (Origene, NM_130830) with a multiplicity of infection of 5. Optimal puromycin concentrations for stable selection of pLenti-LRRC15-GFP-Puro were determined by kill curves. Following selection with puromycin for 14 days, single cell clones were plated by serial dilution, expanded, and sorted for the top 1% of GFP+ cells.

### In vitro studies

#### Pulldown assay (immunoprecipitation - mass spectrometry)

Pierce Protein G Magnetic Beads (ThermoFisher, #88847) were pre-incubated with 70 µg DUNP19 antibody or hIgG1 mAb in PBS at room temperature (RT) for 1 h. Bead-antibody conjugate was recovered using magnetic separation before adding 300 µg protein lysates (isolated from U118MG cells) for 2 h on ice. Beads were washed with PBS and an on-bead digestion was performed with 0.25% Trypsin for 16 h. MS-MS was run using the Agilent 6530 LC/MS in collaboration with the UCLA Molecular Instrumentation Center according to previously described protocols.^35,36^

#### Flow cytometry

To assess the binding affinity of DUNP19, cells were blocked (10% normal goat serum in PBS, 15 min at RT) and incubated with serial dilutions of fluorophore-conjugated DUNP19 or hIgG1 mAb (0.051–1000 ng/mL) in triplicate for 1 h at RT. Cells were washed with 1% BSA in PBS (180 × *g*, 3 min) before adding viability dye per manufacturer’s instructions (Invitrogen, #L34989). Antibody binding capacity of cells was assessed using 1 µg/mL DUNP19 and anti-human IgG Simply Cellular bead standards (Bangs Laboratories, #816). Antigen binding capacity was determined by comparison of mean fluorescent intensity of cell lines to a standard curve generated with IgG Simply Cellular bead standards. Quantity of LRRC15 surface antigens available for DUNP19 binding was normalized to cell surface area, determined experimentally by confocal microscopy. All flow cytometry experiments were run in collaboration with UCLA’s Jonsson Comprehensive Cancer Center Flow Cytometry Shared Resource using an Attune NxT Flow Cytometer (Invitrogen). Flow cytometry data were analyzed using FlowJo (Version 10, BD Biosciences).

#### Reverse transcriptase polymerase chain reaction (RT-PCR)

Expression of *LRRC15* in cells was determined by Taqman qRT-PCR. Cells were lysed and one-step reverse transcription/quantitative PCR performed with Cells-to-CT Taqman kit (A25603). *LRRC15* was probed with Applied Biosystems Taqman Assay probes (Assay ID Hs00370056_s1) and normalized using *GAPDH* housekeeping gene (Assay ID Hs02786624_g1). All qPCR assays were run using the ViiA 7 Real-Time PCR system (Applied Biosystems).

#### Confocal microscopy of cells

For immunocytochemistry, cells were blocked with 10% normal goat serum in PBS for 1 h at room temperature. Cells were stained with DUNP19 (20 µg/mL) and anti-PMCA1 antibody (Abcam, ab3528, 1:100). Primary antibodies were incubated overnight at 4 °C before washing and staining with goat anti-rabbit AlexaFluor647 (1:500, Invitrogen A21235) and goat anti-human AlexaFluor488 (1:500, Invitrogen A11013) secondary antibodies. Cells were fixed with 3.7% paraformaldehyde for 15 min at RT before washing and mounting cells with Vectashield mounting media with DAPI (H1200-10) onto slides for imaging with a Leica TCS SP8 Digital Microscope.

To confirm internalization, cells (0.0015 × 10^6^ cells/well) were seeded in phenol red-free complete media in 384-well u-clear flat bottom black plates (Greiner, #781092) and stained with 1 µg/mL DUNP19-AF647, anti-huLAMP1-A488 antibody (1:250, Invitrogen, 53-1079-42) and Hoechst 33342 (1:2000 dilution, Invitrogen) at 4 °C for 1 h to prevent antibody internalization while promoting surface binding. Unbound antibody was aspirated and replaced with phenol red-free complete media. Time-lapse confocal imaging was performed using a temperature controlled ImageXpress MicroXL High Content Imaging microscope (Molecular Devices). The microscope temperature was set to mimic cell culture conditions (37 °C, 5% CO_2_) and images were taken at 10× magnification (4 sites per well) every 20 min for 6–12 h. The plasma membrane signal of DUNP19-AF647 relative to the cytosolic signal (LAMP1) at time 0 (directly after incubation at 4 °C) was quantified to calculate the fraction of internalized antibodies over time.

### In vivo studies

#### Animal studies

All animal experiments were conducted in compliance with national legislation on laboratory animal protection and permitted by the local ethics committees for animal research at Washington University, Lund University, and University of California, Los Angeles. Mice were housed under pathogen-free conditions in individually ventilated cages, maintained on a 12-h light/dark cycle, with ad libitum access to standard chow and water. Animals were acclimatized for one week prior to the start of the experiments. Mice were randomized according to tumor size. Where feasible, outcome assessments were performed by investigators blinded to group allocation. Mice were euthanized when subcutaneous tumors exceeded 1000 mm³ (15 mm in diameter), when ulceration or bleeding sores developed on the tumor, or when significant overall health decline was observed, including a body weight loss exceeding 20% of baseline.

#### Subcutaneous tumor models

Athymic nude mice (BALB/cAnNRj-Foxn1 nu/nu; 6–8 weeks old, 20–25 g; Janvier) were inoculated with U118MG (5.8 × 10^6^ cells), SAOS2 (6 × 10^6^ cells), HuO9 (6 × 10^6^ cells), K7M2^*LRRC15+*^ (5 × 10^6^ cells), HCC1954 (4.9 × 10^6^ cells) or LNCaP (5 × 10^6^ cells) cells in a 200 μL (1:1 v/v) mixture of media with Matrigel via subcutaneous injection in the right flank. Tumors developed after 3 to 6 weeks. Tumor volume was estimated with caliper measurements twice weekly (V (mm^3^) = 0.5 × length × width^2^).

#### Orthotopic osteosarcoma model

Athymic nude mice (BALB/cAnNRj-Foxn1 nu/nu; males, 6–8 weeks old, 24.2 ± 1 g; Janvier) were anesthetized, and the tibia of the right hind limb was punctured. HuO9 cells (approximately 0.25 × 10^6^ cells in 10 μL media) were injected into the cavity using a microvolume syringe with a 27-gauge needle. Bone wax (Surgical Specialties Corporation, #903) was applied to seal the punctured area and prevent exodus of implanted cells before the area was washed with saline. Tumor development was confirmed using ultra-high-resolution CT and [^177^Lu]Lu-DUNP19 SPECT/CT imaging (nanoScan; Mediso, Budapest, Hungary).

#### Small-animal imaging

PET: Male mice (B6;129-Rag2tm1FwaII2rgtm1Rsky/DwlHsd) (*n* = 5) bearing s.c. SAOS2 xenografts were intravenously (i.v.) injected with [^64^Cu]Cu-DUNP19 (8.75 MBq, 100 µg DUNP19 in 100 μL 10 mM ammonium acetate). Dynamic PET images were acquired during the first hour post-injection (p.i.), followed by static scans (20 min each) at 12 h, 24 h, and 36 h using a microPET R4 rodent scanner (Siemens). To confirm specificity of the DUNP19 PET-signal, imaging with the bone-seeking PET-probe Fluorine 18-sodium fluoride ([^18^F]-NaF; 10 MBq i.v.) was performed in mice bearing SAOS2 tumors. SPECT: Mice with intratibial HuO9 xenografts were i.v. injected with [^177^Lu]Lu-DUNP19 (20 MBq, 30 μg). Mice were scanned for 50–60 min under anesthesia (2–3% isoflurane) using a SPECT/CT device (nanoScan Mediso, Budapest, Hungary). Details on PET, SPECT and CT acquisition are provided in the Supplementary Materials.

#### [^177^Lu]Lu-DUNP19 biodistribution and kinetics

To investigate the biodistribution of [^177^Lu]Lu-DUNP19, mice with s.c. SAOS2, HuO9, U118MG, HCC1954, or K7M2 ^*LRRC15+*^ tumors, respectively, received ca. 0.3–0.5 MBq [^177^Lu]Lu-DUNP19 (SAOS2, U118MG: 8 µg, K7M2 ^*LRRC15+*^: 16 µg; HuO9: 25 µg; HCC1954: 10 µg) by i.v. injection and were sacrificed at predetermined time points (SAOS2: 6, 24, 48, 72, 168, and 336 h p.i.; HuO9: 6, 24, 48, 72, 168, and 336 h p.i.; U118MG: 24, 48, and 72 h p.i.; HCC1954 and K7M2 ^*LRRC15+*^: 72 h p.i.). To confirm targeting specificity, mice bearing LRRC15 + HuO9 and LRRC15- LNCaP (right flank) xenografts were i.v. injected with either [^177^Lu]-hIgG1 mAb or [^177^Lu]Lu-DUNP19 (*n* = 4 mice) and sacrificed 48 h later. To study the effect of the antibody mass amount on the biodistribution and tumor uptake, mice bearing HuO9 tumors were administered escalating DUNP19 doses (0.3, 1, 3, 30, and 100 μg/mouse at 0.5 MBq ^177^Lu; i.v.) and euthanized 72 h p.i. In all studies, blood, tumors (ossification removed where necessary), and several normal tissues were collected, dried and weighed. Data from animals with injection failure or tissue sampling issues were excluded from analysis. The radioactivity contained in the respective tissue and reference standards was quantified in a NaI(Tl) automated well counter (1480 WIZARD; Perkin Elmer). Data were expressed as percent of injected activity per gram tissue (% IA/g).

#### Radiological analysis of tumor calcification

Quantification of radiopacity was analyzed using ImageJ software (version 1.53). A brightness threshold of 41 was used to define the area of each tumor sample. The total area of the tumor was then quantified using ImageJ’s built-in area analysis program. Areas of calcification were defined by a brightness threshold of 81 and quantified in the same manner. Percent calcification was calculated by dividing the area of calcification over the area of the entire tumor.

#### Confocal microscopy of xenografts

Mice with HuO9, SAOS2, and HCC1954 tumors, respectively, were injected with 30 µg DUNP19-AF594 when tumor volume reached between 200 and 300 mm^3^. Sections were washed two times with PBS before permeabilization with 0.1% Triton-X/PBS and blocking for 1 h at RT with 10% goat serum/PBS. Sections were stained with an anti-LAMP1-A488 antibody (HuO9, SAOS2: anti-hLAMP1, 1:250, Invitrogen, #53-1079-42; HC1954: anti-mLAMP1, 1:250, Invitrogen, #53-1071-82, to detect the murine stroma) overnight at 4 °C. The next day, slides were stained with Phalloidin-AF647 for 1 h at RT (1:2000, Invitrogen, #A22283) and mounted with Vectashield Antifade mounting media with DAPI (Vector Laboratories, H-2000-2). Confocal microscopy was done by the UCLA Advanced Light Microscopy and Spectroscopy Laboratory (ALMS) with a Leica TCS-SP8 microscope at 63x magnification and sequential imaging for far-red, orange, green and blue emitting dyes. Z-stack images were taken, and Lightning deconvolution and 3D reconstruction (Leica Microsystems) were performed during post-processing.

#### Therapy studies

Average tumor volume at the start of treatment was 165.81 ± 63 mm^3^ and the average animal weight was 25.2 ± 0.7 g. Mice bearing s.c. HuO9 or U118MG xenografts with LRRC15 expression in both tumor cells as well as tumor stroma, and HCC1954 xenografts with LRRC15 expression only in the tumor stroma, were randomized based on tumor size to receive either [^177^Lu]Lu-DUNP19 (16–30 μg, i.v.) or no treatment (PBS). HCC1954 model: Mice were treated with a single injection of 20 MBq [^177^Lu]Lu-DUNP19. HuO9 model: Animals received three fractionations [^177^Lu]Lu-DUNP19 at days 0, 32, and 75 resulting in a cumulative administered activity of 50 MBq (group 1: 10 + 20 + 20 MBq; group 2: 20 + 10 + 20 MBq). Tumor growth was measured throughout the study. A subset of tumors was imaged using SPECT/CT and µCT to evaluate ossification and the uptake of [^177^Lu]Lu-DUNP19. For efficacy analysis, data beyond day 90—including tumor volumes, survival times, mortality (due to cumulative injections or treatment duration; *n* = 11), and imaging-related inconsistencies—were excluded, with analysis limited to the 90-day endpoint. To investigate the impact of pre-therapeutic tumor volume on the efficacy of [^177^Lu]Lu-DUNP19 in the HuO9 model, mice were left untreated or administered 30 MBq [^177^Lu]Lu-DUNP19 (30 µg, i.v.) when tumors reached a volume of 171 ± 56 mm^3^ (group 1) or 470 ± 121 mm^3^ (group 2). Group 1 was injected on day 19, Group 2 on day 40 post inoculation. Mice were excluded for no tumor (*n* = 1), out-of-range tumor volumes (*n* = 2, Group 1; *n* = 5, Group 2), or death/weight loss prior to day 40 (comparison point) (*n* = 3, Group 1). To minimize animal use, a common untreated control group was used, with analyses conducted separately for each study. U118MG model: Mice were treated with two fractions [^177^Lu]Lu-DUNP19 at days 0 and 34 and a cumulative activity of 20 MBq (10 + 10 MBq) or 30 MBq (20 + 10 MBq). Treatment efficacy was assessed by measuring tumor growth and time to a humane endpoint. Survival was analyzed by using a log-rank test in GraphPad Prism. *P* values < 0.05 were considered significant statistically. Tumors from a subset of mice were harvested 62–116 days p.i. (except U118MG: day 155–190 p.i.) and processed for RNA-sequencing (see below).

To evaluate the efficacy of [^177^Lu]Lu-DUNP19 in a clinically relevant orthotopic osteosarcoma model, mice with intratibial HuO9 tumors were randomized (23 days post-tumor engraftment) to receive 20 MBq [^177^Lu]Lu-DUNP19 (30 µg, i.v.) or PBS. Four days after treatment, [^177^Lu]Lu-DUNP19 tumor uptake and presence of viable HuO9 tumor was assessed by SPECT/CT imaging. At day 163 post-first injection, mice received an additional 20 MBq [^177^Lu]Lu-DUNP19 and were re-scanned to detect residual viable HuO9 tumor tissue. Mice were followed up for 190 days after the first [^177^Lu]Lu-DUNP19 injection.

To evaluate the antitumor effects of combination therapy in a syngeneic mouse model, female 6–8-week-old C57Bl/6 animals (Inotiv) were implanted with 0.5 M MC38-Luc cells, subcutaneously, in the right flank. Tumor volume and animal weight was monitored 2–3 times per week for the duration of the study. Animals were treated with unconjugated DUNP19, combination immune checkpoint inhibition (anti-CTLA4 and anti-PD-1), [^177^Lu]Lu-DUNP19, or the combination of immune checkpoint inhibition and radiotherapy. Anti-CTLA4 (9D9) and anti-PD-1 (RMP1-14) antibodies were purchased from Leinco. Antibodies (100 µg, in saline) were intraperitoneally administered on three consecutive days after average tumor volume reached 200 mm^3^. Animals received unconjugated DUNP19 (30 µg) in the control group or [^177^Lu]Lu-DUNP19 (7.4 MBq; 30 µg) one day after the first injection of immune checkpoint inhibition.

#### Hematological toxicity of [^177^Lu]Lu-DUNP19

Hematological toxicity, recovery, and body weight were monitored in mice treated with [^177^Lu]Lu-DUNP19. Blood samples were taken before and weekly after injection of [^177^Lu]Lu-DUNP19 for 4 weeks p.i. Samples (20 µL) were collected from the tail vein of awake, immobilized mice by piercing the vein with a needle (27 G) and collecting blood in a K2EDTA-coated plastic micropipette. Blood cell counts were obtained using an Exigo Veterinary Hematology Analyzer (Boule Medical, Stockholm, Sweden).

#### Gene expression analysis

For RNA-sequencing, tumor tissues were harvested at 62–116 days (HuO9, HCC1954) or 155–190 days (U118MG) post-treatment, apart from untreated mice (harvested when tumor volume measured greater than 1000 mm^3^, in accordance with established endpoint protocols). Tumor tissue was preserved in RNA*later* stabilization solution (Invitrogen, AM7020) before RNA isolation with the Qiagen RNeasy kit (#74004). RNA quality control was assayed via TapeStation (Agilent) and stranded mRNA library preparation performed in accordance with Illumina protocols. Samples were sequenced on Illumina’s Novaseq platform to generate 50 bp single reads. Library preparation and sequencing was done with the help of UCLA’s Technology Center for Genomics and Bioinformatics (TCGB).

Raw read count RNA-sequencing data were generated from untreated and [^177^Lu]Lu-DUNP19 treated HuO9 tumors. Reads were aligned to either human (Hg38) or murine (Mm19) genome using the STAR method, as previously described.^37^ Ambiguous reads were discarded and FastQC analysis was utilized to confirm sequence quality. Low read count filtering was used to remove transcriptomic features for which fewer than 4 samples had at least 5 read counts of a gene, as described by the EdgeR differential analysis user guide.^38^ For each tumor model, principal component analysis based on log2 counts in RStudio Version 2023.06.1 was plotted. K-Clustering and heat maps were generated on log2-transformed read counts to visualize gene signatures in treated versus untreated samples. Differential expression analysis to identify differentially expressed genes was performed using EdgeR (Bioconducter, Version 3.40.2) using quasi-likelihood F-tests within the EdgeR program. A false discovery rate of 0.05 (adjusted using Benjamini-Hochburg methodology) and absolute log2-fold change >1 were selected as the cutoff for DEGs within this analysis. A positive fold-change represented upregulation and a negative fold change represented downregulation of gene expression in treated tumors. For comparison and visualization of gene expression between clustered samples, z-scores were calculated per gene. Pathway analysis was performed using gene set enrichment analysis and molecular signatures defined using the human and murine Molecular Signature Database Hallmark pathways.^39^ Additional analysis was performed using Gene Ontology (GO) Biological Pathways. For cell classification and identification, Syllogist^22^ was used to identify signatures present from 43 cell types within normalized gene expression matrices. For murine cell classification, murine orthologs were matched to Syllogist cell signatures using g:Profiler.

#### Statistical analyses

Statistical analyses were conducted using Graphpad Prism software (Version 9.5.1). Data are expressed as mean and standard deviation. Tumor volumes are presented as mean ± SD. Statistical comparisons were performed using one-way ANOVA with Bonferroni’s multi-comparison tests, Log-rank (Mantel-Cox) test, and unpaired Student’s t-tests. A *P*-value of less than 0.05 was considered statistically significant.

## Supplementary information

Supplementary Materials

## Data Availability

RNA-sequencing data are available from the Gene Expression Omnibus NCBI database under accession number GSE260644. All data supported in the conclusions are included in the manuscript and supplementary materials.
