# Supplementary material for: Development of a leucine-rich repeat-containing protein 15-targeted radio-immunotheranostic approach to deplete pro-tumorigenic mechanisms and immunotherapy resistance
Source: Signal Transduct Target Ther. 2025 Sep 30;10:319. doi: 10.1038/s41392-025-02410-9 (PMC12480655; doi:10.1038/s41392-025-02410-9)
Supplement: Supplementary file 1 — Supplementary Materials [file 41392_2025_2410_MOESM1_ESM.docx]

Supplementary Materials for

**Development of a LRRC15-targeted radio-immunotheranostic approach to deplete pro-tumorigenic mechanisms and immunotherapy resistance**

Claire M. Storey*, Mohamed Altai*, Katharina Lückerath, Wahed Zedan, Henan Zhu, Lara Breuer, Marija Trajkovic-Arsic, Julie Park, Abbie Hasson, Jens Siveke, Diane Abou, Haley Marks, Enna Ulmert, Alexander Ridley, Marcella Safi, Urpo Lamminmäki, Constance Yuen, Susanne Geres, Liqun Mao, Michael Cheng, Sumit K. Subuhdi, Bilal A. Siddiqui, Noah Federman, Johannes Czernin, Ken Herrmann, Laurent Bentolila, Xia Yang, Thomas G. Graeber, Robert Damoiseaux, Daniel Thorek, and David Ulmert

Correspondence to: HUlmert@mednet.ucla.edu

**This PDF file includes:**

Materials and Methods

Supplementary Figures S1 to S13

Supplementary Tables S1 to S3

**Supplementary Methods**

Determination of Antibody Binding Kinetics and Antigen Affinity

The binding affinity of DUNP19 for LRRC15 was assessed using biolayer interferometry (BLI) on an Octet RED384 system (Sartorius). DUNP19 was biotinylated by incubation with a 20-fold molar excess of EZ-Link NHS-PEG4-biotin (Thermo Fisher) in phosphate-buffered saline (PBS, pH 7.4) for 30 min at room temperature, followed by buffer exchange using NAP-5 and NAP-10 columns to remove unreacted biotinylation reagents. For kinetic analysis, the biotinylated DUNP19 (0.8 µg) was immobilized onto streptavidin-coated sensor tips and exposed to recombinant extracellular domains of human and mouse LRRC15 (AcroBiosciences) at a range of analyte concentrations. Human LRRC15 was tested at 50 pM to 50 nM, while mouse LRRC15 was assessed at 100 nM to 600 nM. Association and dissociation kinetics were determined, and equilibrium dissociation constants (KD) were derived from global fitting of the binding curves. The KD values for human and mouse LRRC15 were determined to be 175 pM and 370 nM, respectively, indicating a higher affinity for the human ortholog.  Despite the significant difference in affinities, DUNP19 demonstrates efficient binding to LRRC15-overexpressing human cancer cells used in xenografts and murine LRRC15+ cancer-associated fibroblasts (CAFs). The disparity in affinities appears less pronounced in vivo, which may stem from the limitations of using recombinant LRRC15 to assess binding affinity. Recombinant proteins, while useful for controlled studies, often fail to fully recapitulate the conformational and contextual properties of membrane-bound proteins as they exist on the cell surface. For instance, recombinant LRRC15 may lack critical post-translational modifications, such as glycosylation, or the native structural constraints imposed by the lipid bilayer, both of which can influence epitope presentation and binding dynamics. In contrast, assessing affinity using cells expressing LRRC15 preserves these physiological features, providing a more accurate representation of how DUNP19 interacts with its target in a biological context. The downside of relying solely on recombinant proteins is that affinity measurements may overestimate or underestimate true binding strength, potentially leading to discrepancies when translating findings to in vivo systems.

Additionally, the high avidity of LRRC15 when expressed on the cell surface likely enhances the apparent affinity of DUNP19 for its target. Avidity refers to the cumulative binding strength resulting from multiple interactions between an antibody and a densely clustered target, as opposed to the intrinsic affinity of a single binding site. On a cell surface, LRRC15 molecules may be presented in close proximity, allowing DUNP19 - an antibody or similar multivalent binder—to engage multiple LRRC15 units simultaneously. This cooperative binding amplifies the overall interaction strength, compensating for lower intrinsic affinity and improving target retention. This avidity-driven enhancement is particularly relevant in vivo, where the spatial organization of LRRC15 on cancer cells or CAFs could facilitate more effective targeting compared to the isolated recombinant protein scenario.

Regardless of these considerations, the potential for tumor stroma targeting in humans remains promising. The efficient binding observed in preclinical models suggests that DUNP19 could translate effectively to human applications, where the native expression and organization of LRRC15 in the tumor microenvironment may further optimize therapeutic outcomes.

Conjugation and radiolabeling of DUNP19

All buffers were treated with Chelex 100 resin (sodium form, Merck KGaA, Darmstadt, Germany) to remove any metal ions and filtered through a 0.22 μm filter before use. Prior to conjugation, DUNP19 was buffer exchanged using an AMICON Ultra-0.5-centrifugal filter device with a MWCO 30 kDa (Millipore, Burlington, MA, USA). DUNP19 (500 μg in 500 μl 0.07 M sodium borate, pH 9.3) was mixed with the bifunctional chelator p-SCN-CHX-A’’-DTPA (Macrocyclics, Texas, USA) in a 6:1 molar ratio. The mixture was extensively vortexed and incubated overnight at 38°C. The reaction mixture was then centrifuged for 10 minutes at 14000 x g using a 30 kDa filter to remove the excess non-bound chelator. The concentrate containing the CHX-A’’-DTPA-DUNP19 conjugate was recovered, and the buffer was adjusted to 0.2 M ammonium acetate (pH 5.5). The conjugate was aliquoted and kept at −20 °C until labeling. For labeling with ^177^Lu, 30-50 μg of the conjugate was mixed with 15-20 MBq ^177^LuCl3 (Curium, Sweden) and incubated at 38°C with continuous vortexing for 30 min. Thereafter the radiolabeled conjugate was purified, and buffer exchanged using AMICON 30 kDa filter. Radiochemical yield and purity of the radioconjugate were determined using silica-impregnated ITLC strips (150–771 DARK GREEN Tec-Control Chromatography strips, Biodex Medical Systems) eluted with 0.2 M citric acid and measured using the Cyclone Storage Phosphor System (PerkinElmer, Waltham, MA, USA). To reduce radiolysis, the final product was diluted with 1% BSA/PBS buffer (pH 7.4) and 100X molar excess of EDTA was added to scavenge any free metal ions. To determine the maximal attainable specific activity for labeling DUNP19 with ^177^Lu, decreasing amounts of CHX-A’’-DTPA-DUNP19 (10, 5, and 2.5 g) were incubated at 38°C for 30 minutes with a fixed quantity of ^177^LuCl3 (10 MBq). The radiochemical yield was determined using SG-ITLC, as previously described.

Conjugation of p-SCN-Bn-NOTA chelator to DUNP19 was performed in a manner similar to that used in the CHX-A’’-DTPA conjugation described above. Copper-64 was produced at the Washington University in St. Louis School of Medicine Cyclotron facility. ^64^CuCl2 (20 mCi; 25 uL) was diluted with a 10-fold excess 0.1 M ammonium acetate (NH4OAc), pH 5.5 and then added to NOTA-conjugated anti-LRRC15 antibody. After mixing for 30 min at room temperature, the antibody conjugate was purified by gel chromatography (PD10) into 0.1 M HEPES buffer in saline. Purity was assessed by radioITLC (Bioscan AR2000 using samples spotted on Whatman paper in a running buffer of 50 mM DTPA (pH 5.5). Quantitative labeling with >99% radiochemical purity was observed.

SPECT/CT imaging
In vivo SPECT/CT imaging was performed using a nanoScan SPECT/CT system (Mediso). Mice were anesthetized with 2.5% isoflurane and imaged 72 hours after administration of [¹⁷⁷Lu]Lu-DUNP19. CT scans of the same region were acquired to provide an anatomical reference and for attenuation correction. Quantitative image analysis was performed using VivoQuant 3.0 software (inviCRO).

**Supplementary Figures**

**a b**

**
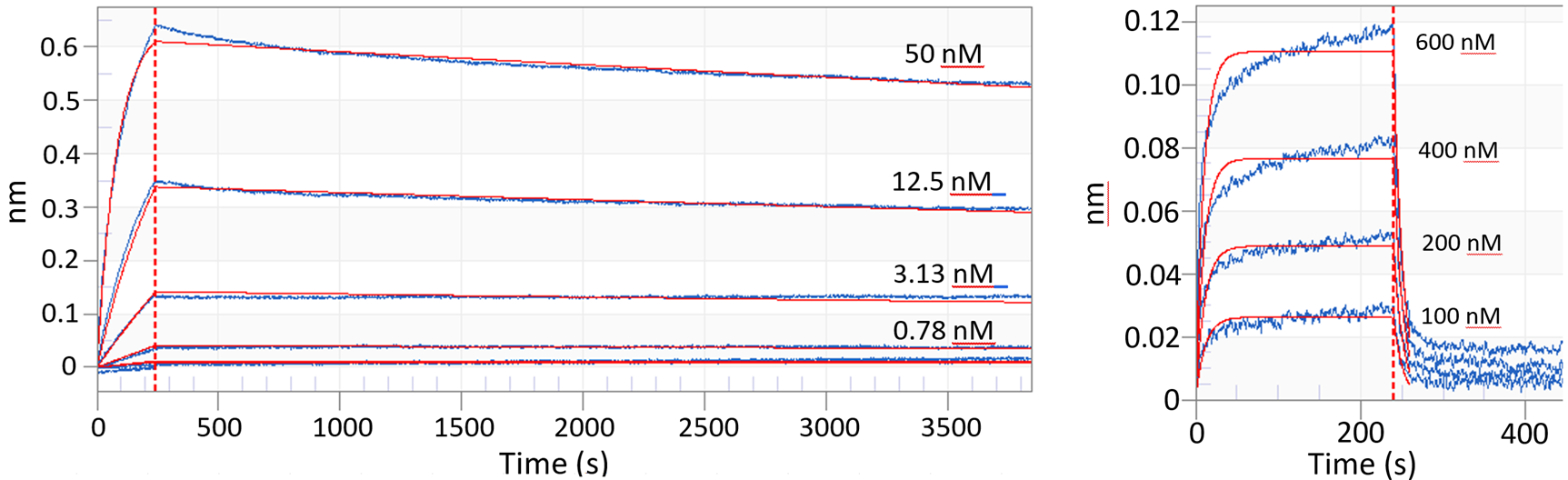
**

**c**


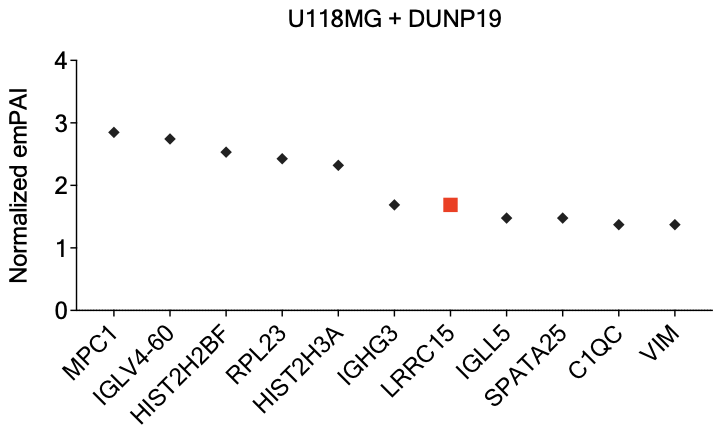


Supplementary Figure 1.

DUNP19 binds to human and murine LRRC15 protein. Biolayer interferometry response curves for the interaction between DUNP19 and LRRC15. For determination of K_D,_ the association and dissociation kinetics of immobilized DUNP19 were analyzed with a range of concentrations of the recombinantly produced extracellular domains of human (a.) and mouse (b.) LRRC15 . **c.** The 11 unique proteins present after an immunoprecipitation-mass spectrometry (IP-MS) analysis of crude protein lysates from U118MG cells incubated with Protein G magnetic bead-conjugated DUNP19. Pulldown-MS composition was compared to protein composition present in non-specific IgG1 with U118MG lysates (n=3). LRRC15 is among the top proteins in complex with DUNP19.


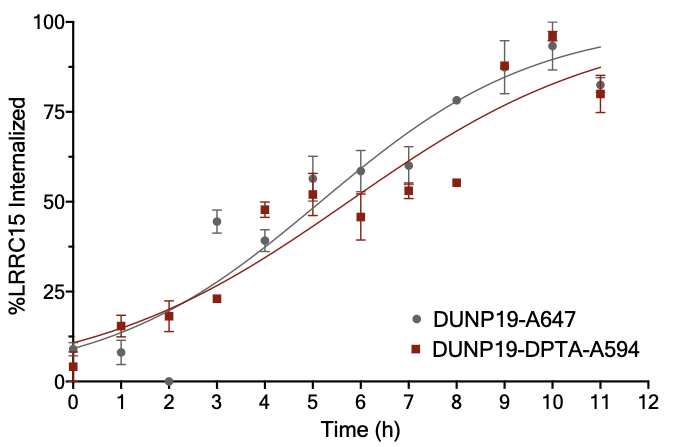


Effect of chelator on DUNP19 internalization (U118MG)

**a**

**b**

Internalization of DUNP19 in fibroblast cells (Hs819.T)


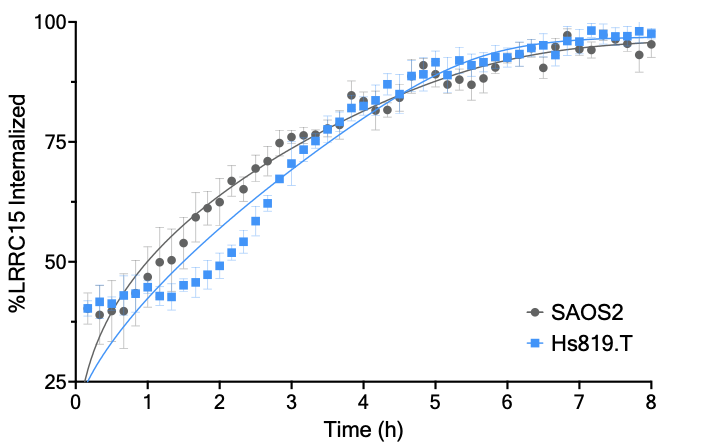


Supplementary Figure 2.

Live confocal microscopy-based assays to determine internalization kinetics of DUNP19 after chelator conjugation and in various cell types. **a.** AF647-DUNP19 or AF647-DUNP19-DOTA conjugate internalize into U118MG cells at similar rates. To determine internalization rate, images were taken every hour across 12 h at 37C. Time to 50% internalization (T_1/2_) of AF647-DUNP19 (measured by ratio of cytosolic intensity of AF647-DUNP19 compared to membrane integrated intensity) was 5.16 ± 0.83 h. The AF647-DUNP19-DOTA conjugate internalized at a similar rate, with a T_1/2_ of 5.75 ± 0.93 h (n=3-4). **b.** Internalization of AF647-DUNP19 in fibroblast-derived Hs819.T cells compared to osteosarcoma SAOS2 cancer cells. Internalization rate was determined by an 8 h microscopy-based assay. Images were taken every 10 min at 37ºC. Time to 50% internalization (T_1/2_) of AF647-DUNP19 in SAOS2 cells was 1.31 ± 0.14 h. T_1/2_ internalization of AF647-DUNP19 into Hs819.T cells was significantly slower with a T_1/2_ of 1.66 ± 0.09 h (n=3-4).

**
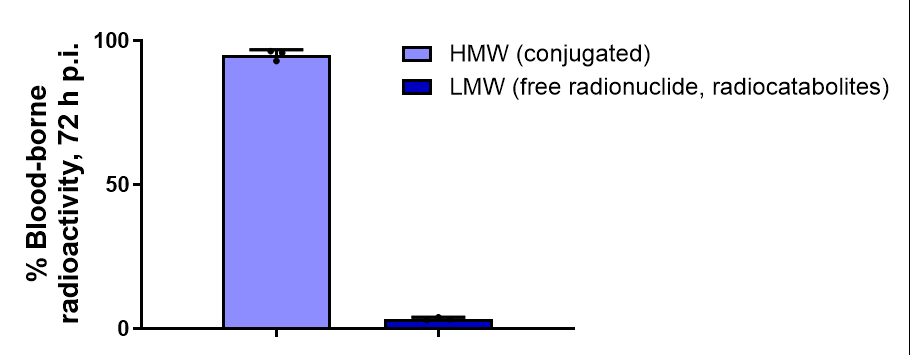
**

Supplementary Figure 3.

Stability of the therapeutic agent [^177^Lu]Lu-DUNP19. To analyze the percentage of radiometabolites in blood samples (n=3) collected 72 hours post-antibody administration, plasma was separated from whole blood through centrifugation at 30,000 rpm for 10–15 minutes at 4°C. The plasma was then processed using size-exclusion NAP-5 columns with a 5 kDa molecular weight cutoff, pre-equilibrated with 1% albumin in PBS, to separate high molecular weight (HMW, >5 kDa) components from low molecular weight (LMW, <5 kDa) components. Radioactivity was measured in each fraction using a gamma spectrometer, and the percentage of radioactivity associated was calculated. The majority of radioactivity in the blood was retained in the HMW fraction, corresponding to the antibody conjugate.

**
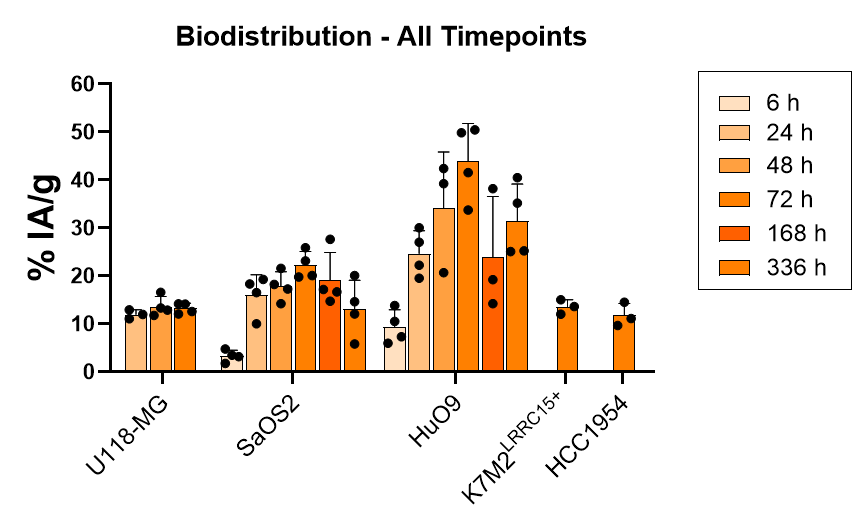
**

Supplementary Figure 4.

Expanded tumor biodistribution of [^177^Lu]Lu-DUNP19 in U118MG, SAOS2, HuO9, K7M2*^LRRC15+^* and HCC1954 xenograft models across 6 timepoints; 6h, 24h, 48h, 72h, 168h, and 336h. Tumor accumulation of [^177^Lu]Lu-DUNP19 peaks between 48-72 h for all models and is represented as %IA/g (percent injected activity per gram tissue).


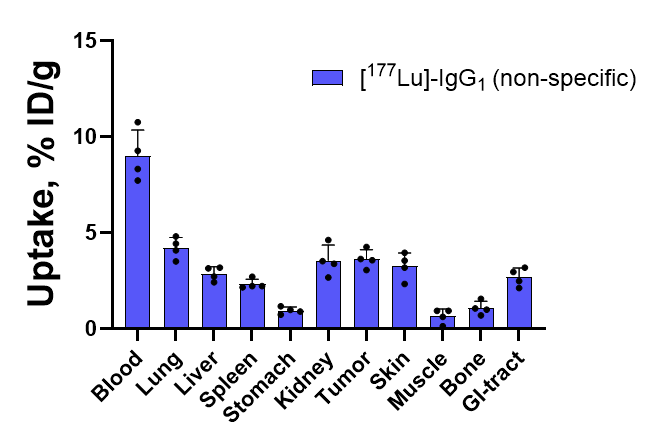


Supplementary Figure 5.

Mice bearing LRRC15+ HuO9 xenografts were i.v. injected with [^177^Lu]Lu-hIgG1 mAb (n=4 mice) and sacrificed 48 hours later. Blood and normal tissues were collected, dried and weighed before quantification of radioactivity.

U118


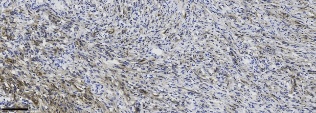

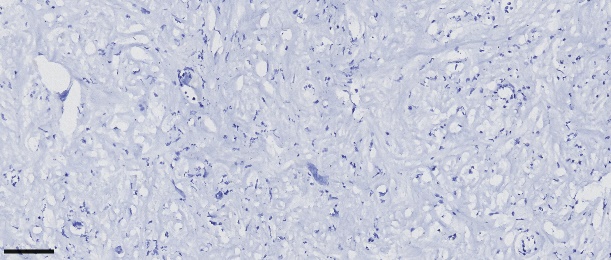

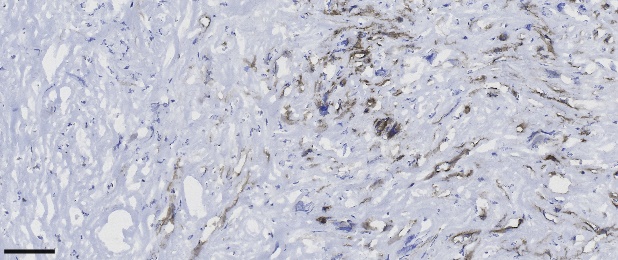

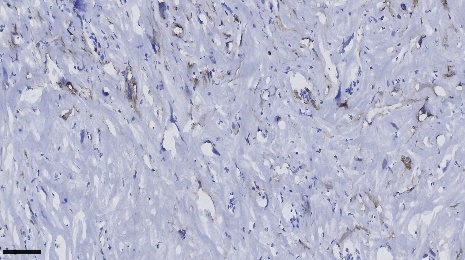


HCC1954

HU09

Vehicle

Treated


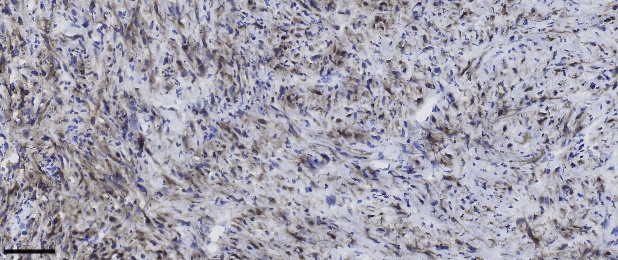

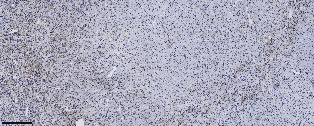

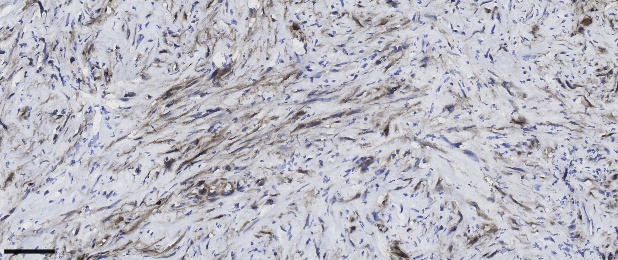

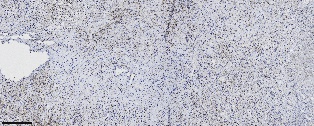

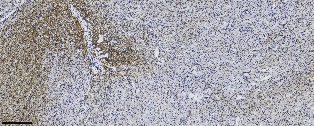

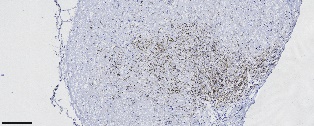

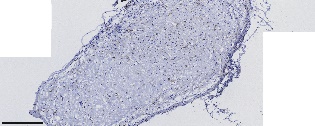

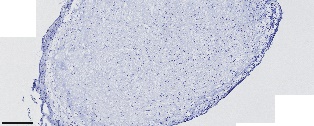

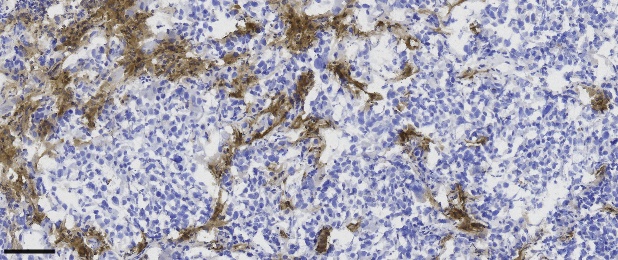

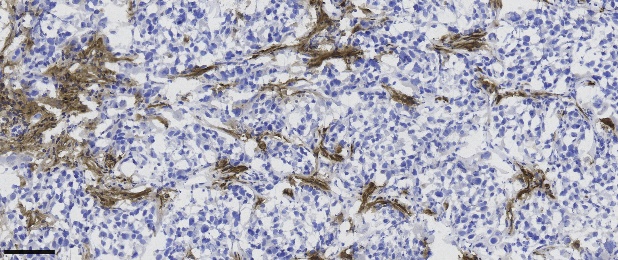

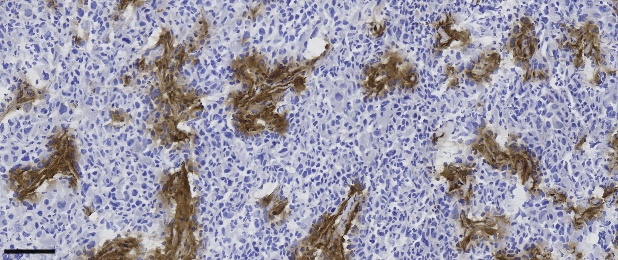

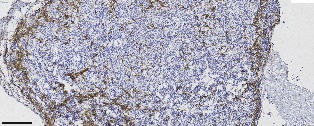

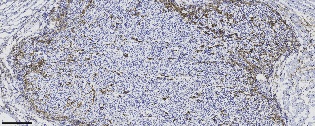

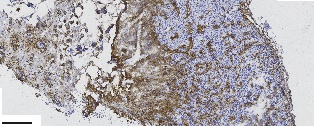

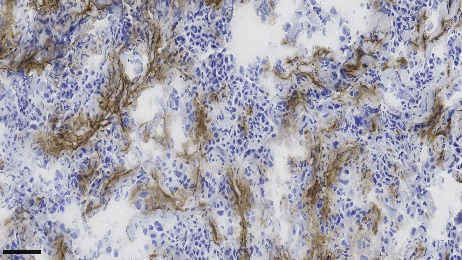

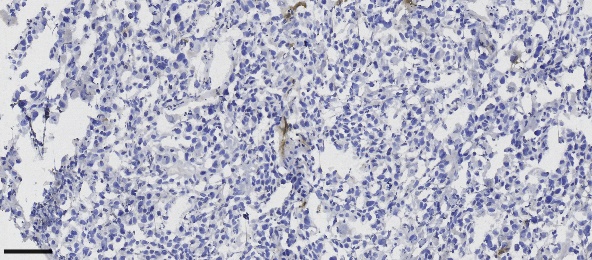

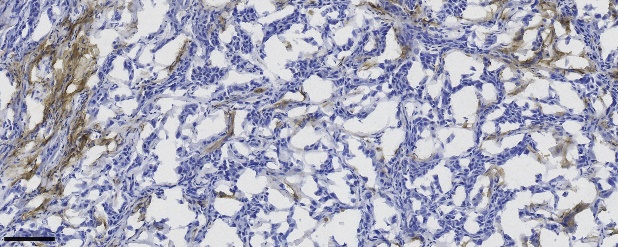

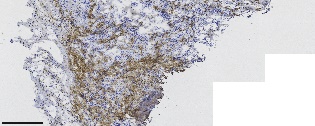

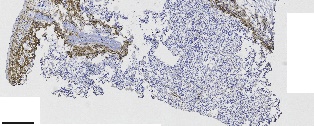

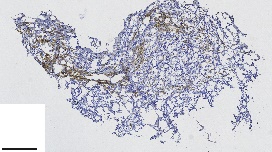

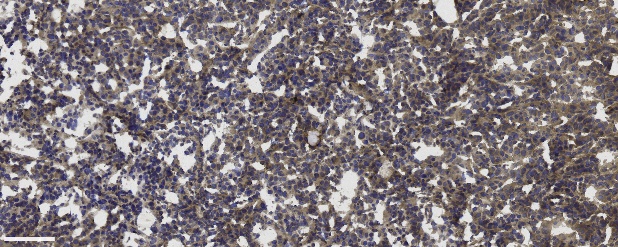

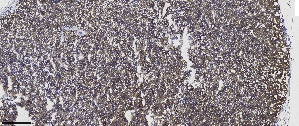

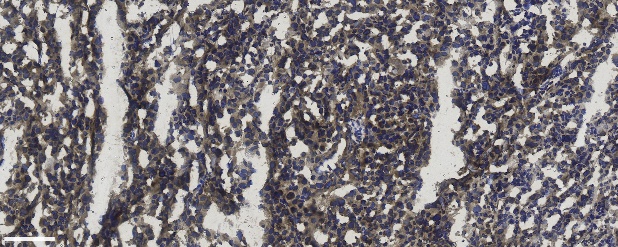

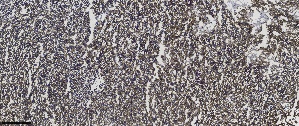

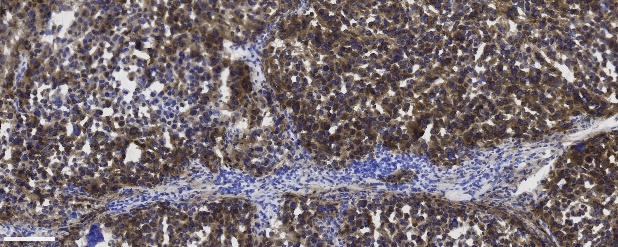

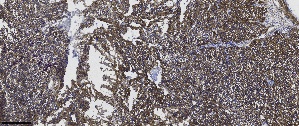

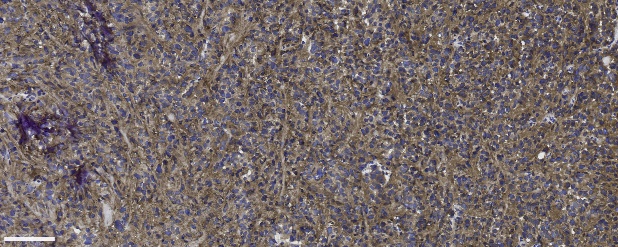

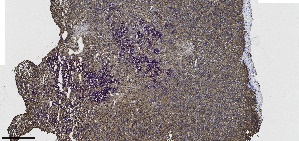

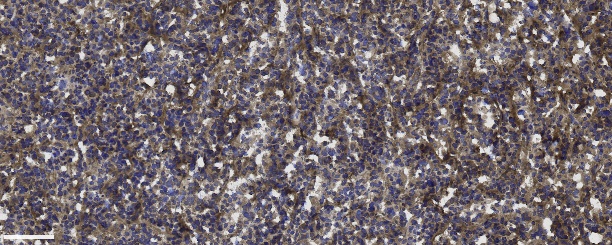

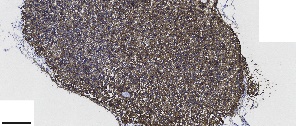

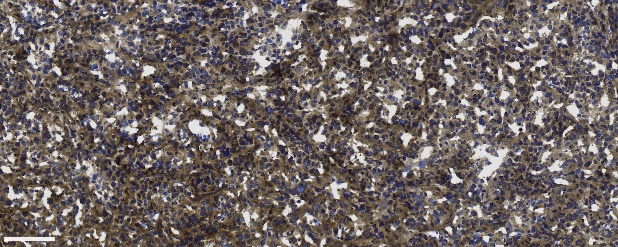

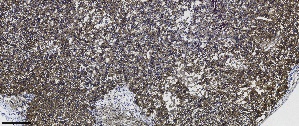


Technical control – secondary antibody only


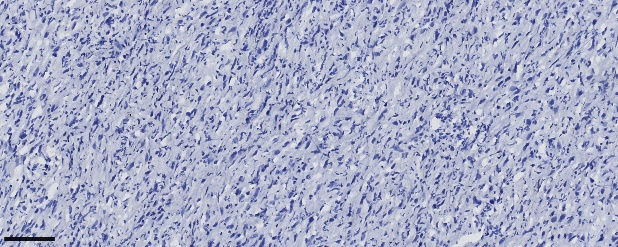

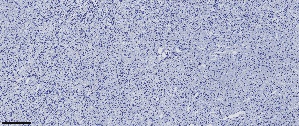


Scale bar: 100 µm

Small overview: 500 µm

Supplementary Figure 6.

Expression patterns of LRRC15 (brown) in IHC images on paraffin-embedded tissues from U118, HCC1954 and HU09 tumor sections (10 µm). Scale bar 100 µm; insert: 500 µm. Three representative tumor sections per group are shown. For the technical control (far right), staining with the primary antibody was omitted.

SAOS2



DAPI

DUNP19

ACTIN

LAMP1 (HUMAN)


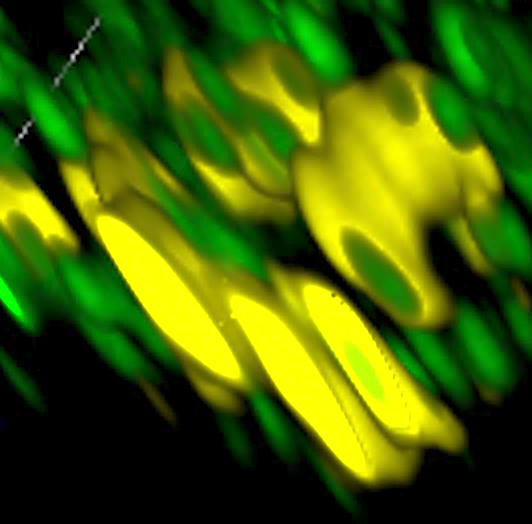

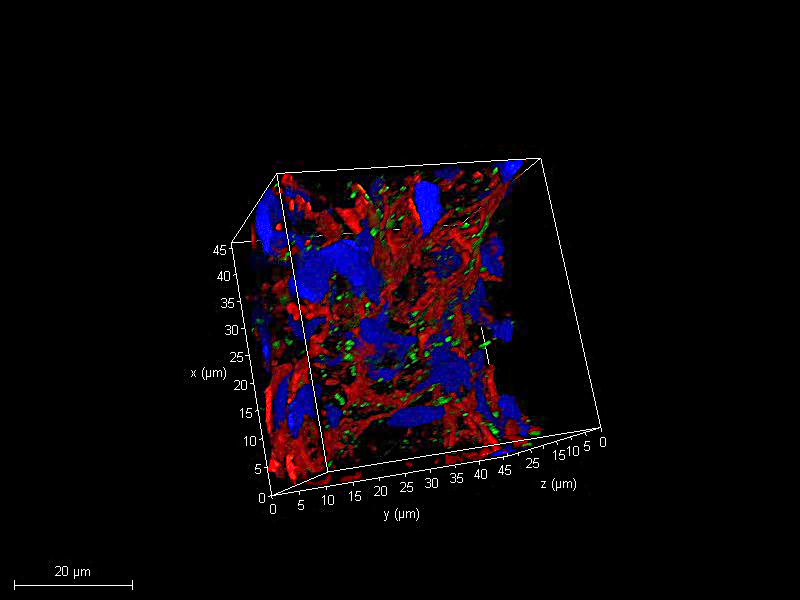

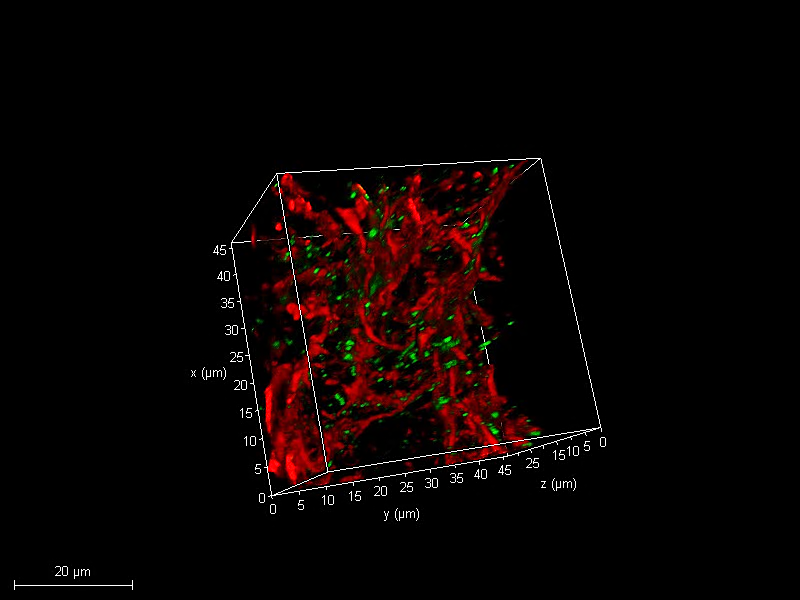

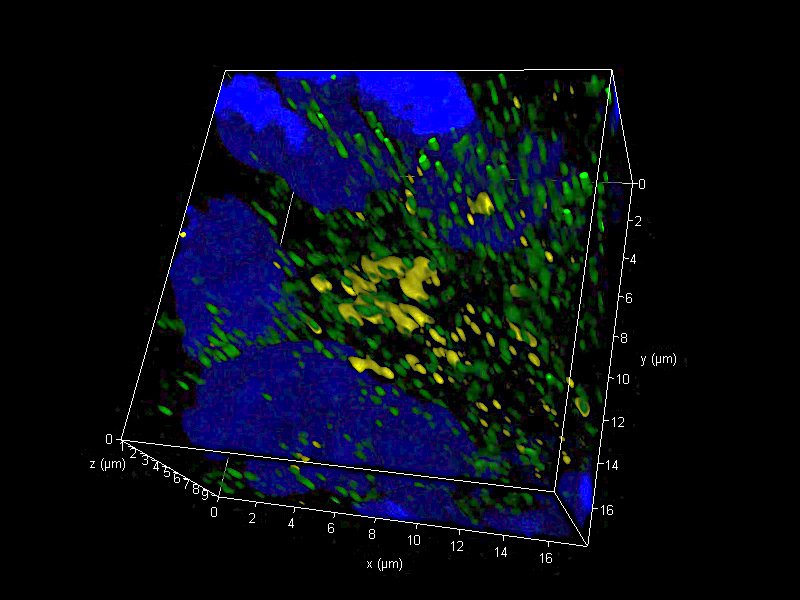

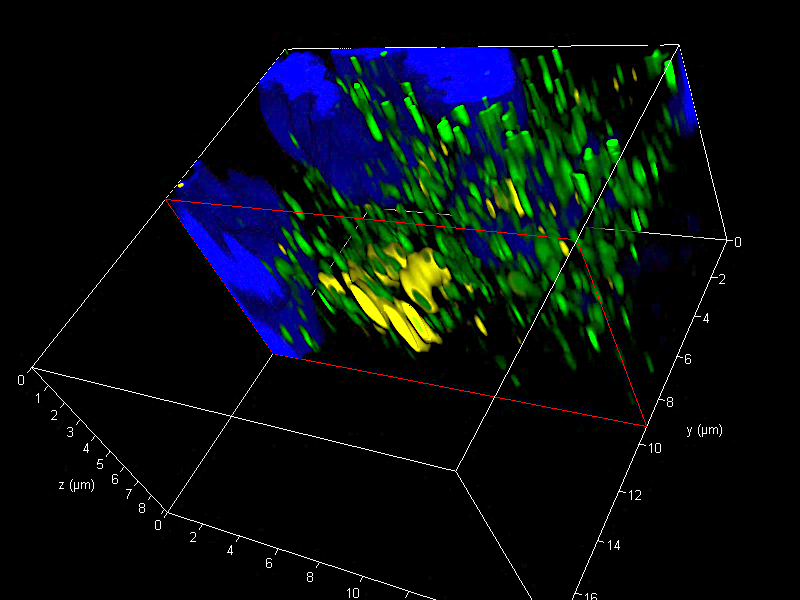


20um

Supplementary Figure 7.

Confocal microscopy of tumor tissues obtained from animals treated with fluorescently labeled DUNP19. Confocal images of s.c. SAOS2 (LRRC15+ cancer cells / LRRC15+ CAF) tumors harvested at 72 h post- i.v. injection of AF594-DUNP19 (yellow). Tumor sections were co-stained for Actin (red), DNA (DAPI, blue) and LAMP1 (lysosomal marker, green) (n=2). Images show that DUNP19 accumulates in the cellular cytoplasm and co-localized with LAMP1 indicating intracellular trafficking of the mAb to the lysosomal compartments (arrow) after binding to LRRC15.


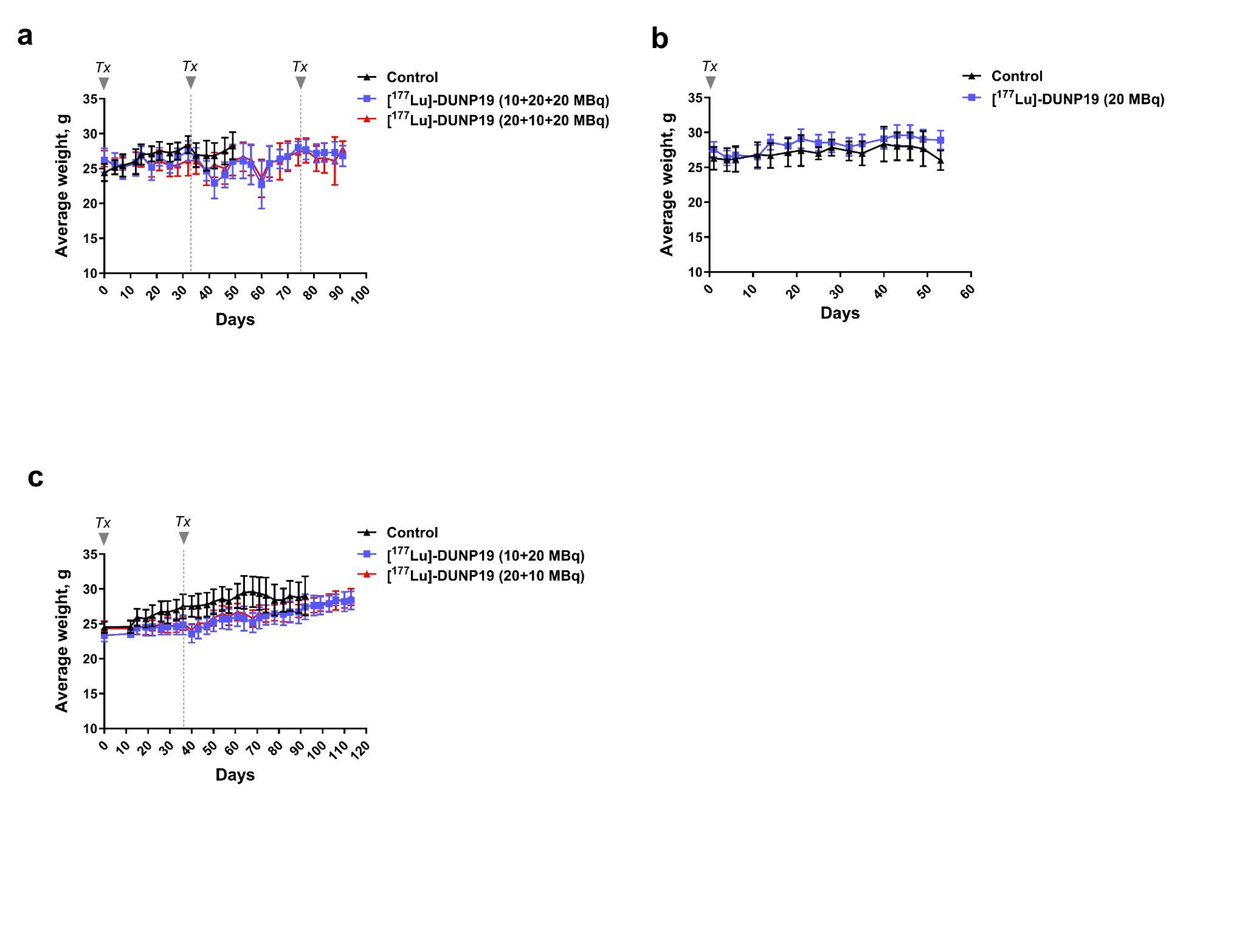


Supplementary Figure 8.

Animal weights across tumor models (HuO9, U118MG, HCC1954) corresponding to therapy studies in Figure 3 (HuO9, top), and Figure 4 (U118MG, middle, and HCC1954, bottom). Gray arrows denote administration of [^177^Lu]Lu-DUNP19 or PBS. Overall, weight was stable throughout [^177^Lu]Lu-DUNP19 therapy studies across tumor models.

**
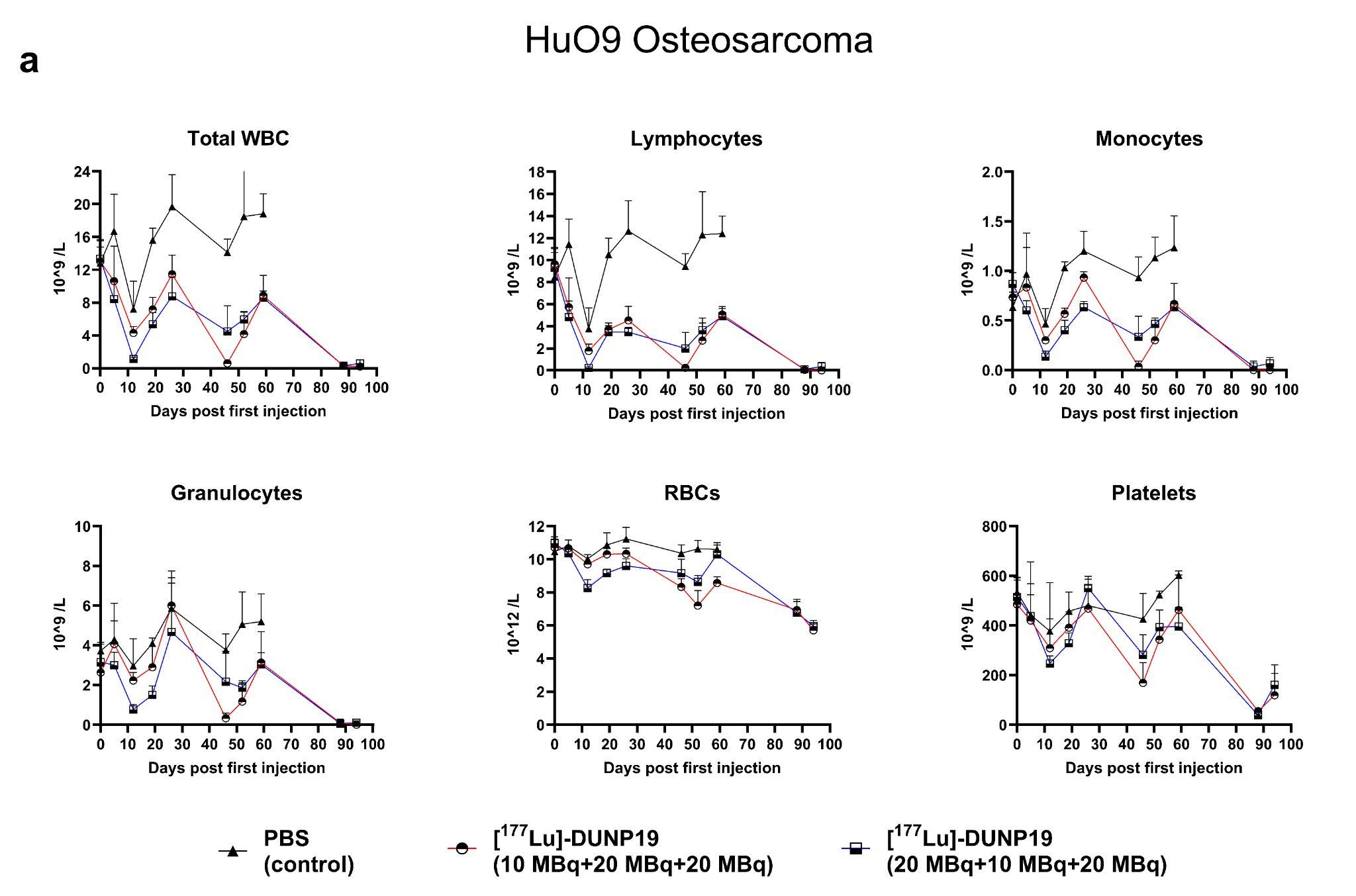
**

**
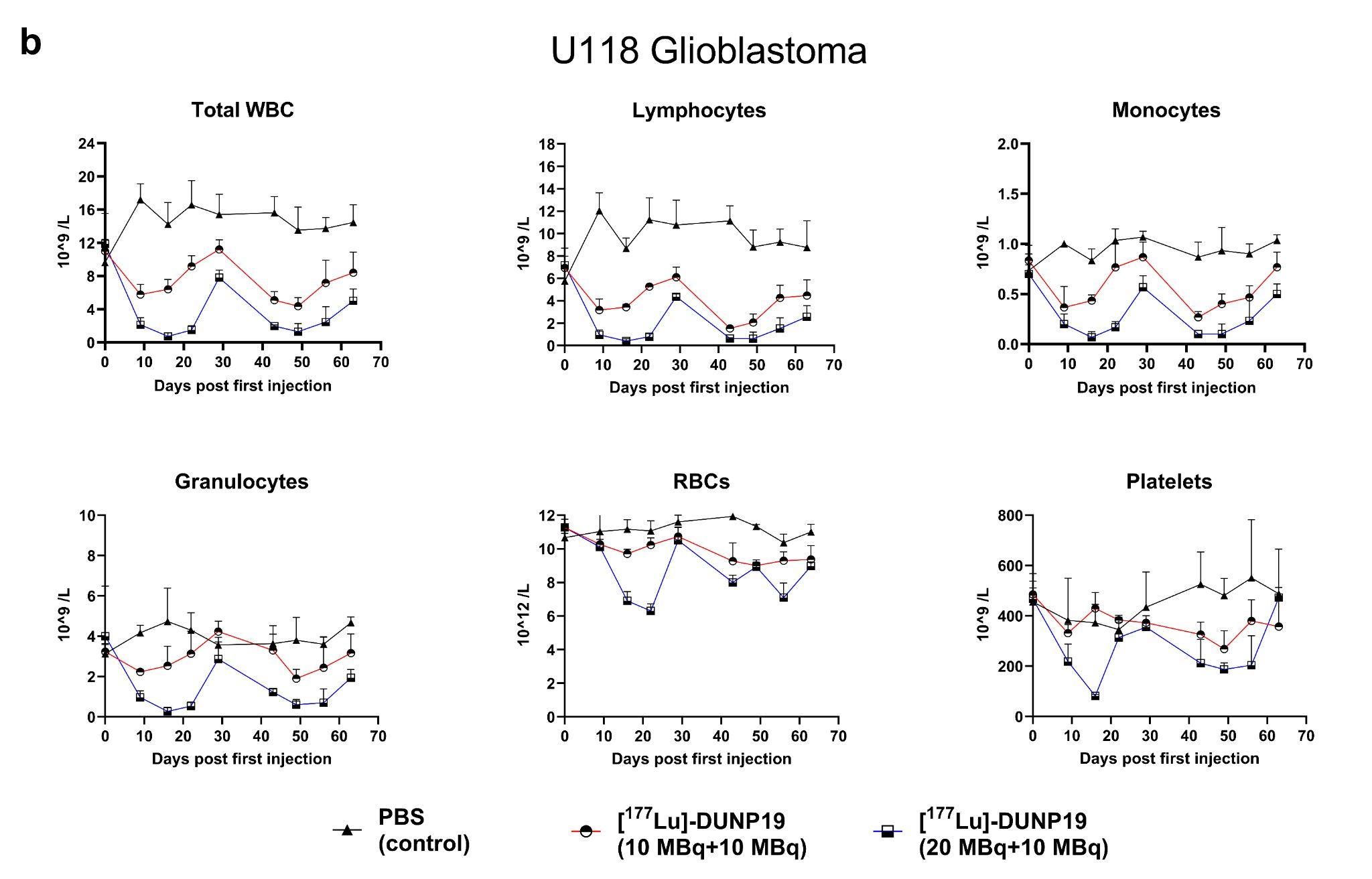
**

**
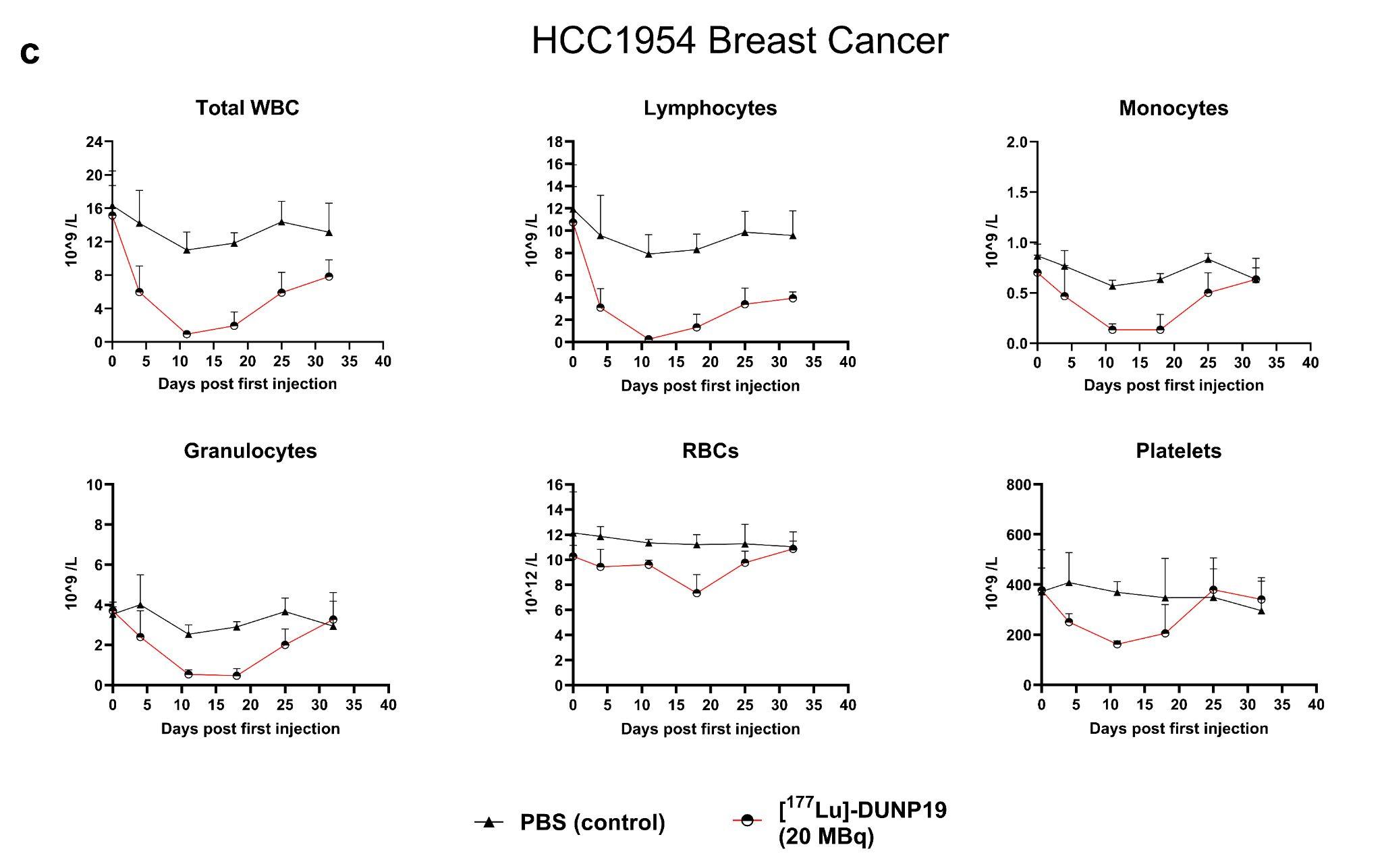
**

Supplementary Figure 9.

Hematological studies examining blood cell counts during treatment course across tumor models (HuO9, U118MG, HCC1954). HuO9 (top), U118MG (middle) and HCC1954 (bottom) exhibited transient reductions in lymphocytes and monocytes after administration of [^177^Lu]Lu-DUNP19, which recovered to baseline levels within 3 weeks, recapitulating toxicity profiles observed in clinical cases.


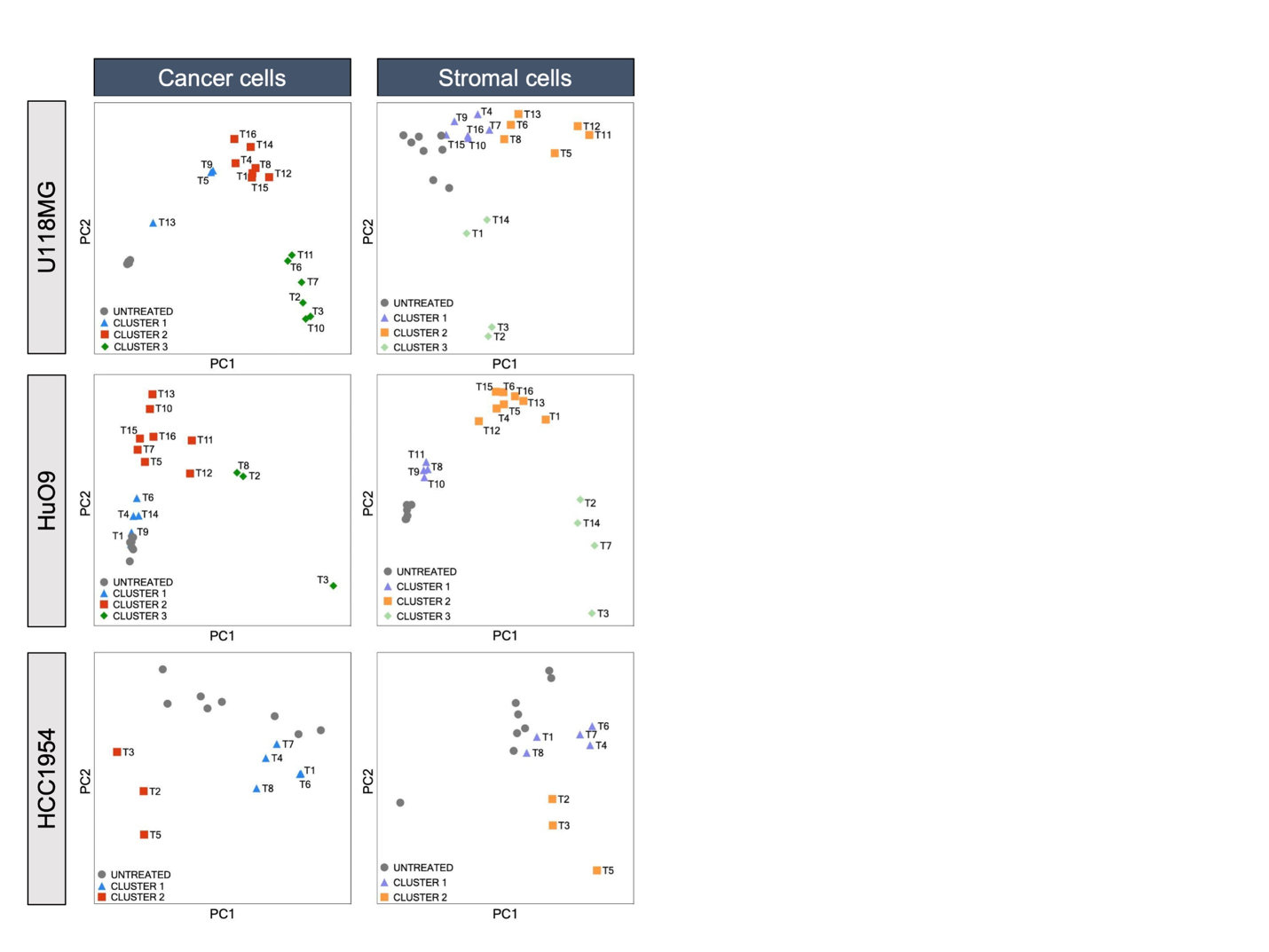


Supplementary Figure 10.

Principal component analysis (PCA) plots of whole transcriptome cancer cell reads (left) and stromal cell reads (right). PC1 and PC2 show separation of treated samples in distinct clusters that were determined to correlate with LRRC15 transcript responses to [^177^Lu]Lu-DUNP19 RIT. In HuO9 and HCC1954 tumor models, treated cancer cells and stromal cells from the same sample clustered in similar patterns. U118MG and HuO9 treated tumors were grouped into three distinct clusters, whereas HCC1954 treated tumors formed two clusters that were differentiated from untreated mice.

[https://mts-sigtrans.nature.com/cgi-bin/main.plex?el=A2Tj5GSa3C6nhe4F1A9ftdcfP99PeAL91S5uS01ltTAZ](https://urldefense.com/v3/__https:/mts-sigtrans.nature.com/cgi-bin/main.plex?el=A2Tj5GSa3C6nhe4F1A9ftdcfP99PeAL91S5uS01ltTAZ__;!!F9wkZZsI-LA!DT29pz3dSm6H2IS3ZfCRZMRSPD-YJ72Q5y1u0JfyA9o3K8aKTj6gLA-38WihRCZOejUhRBnpkWyZRNf8c4uN2A$)


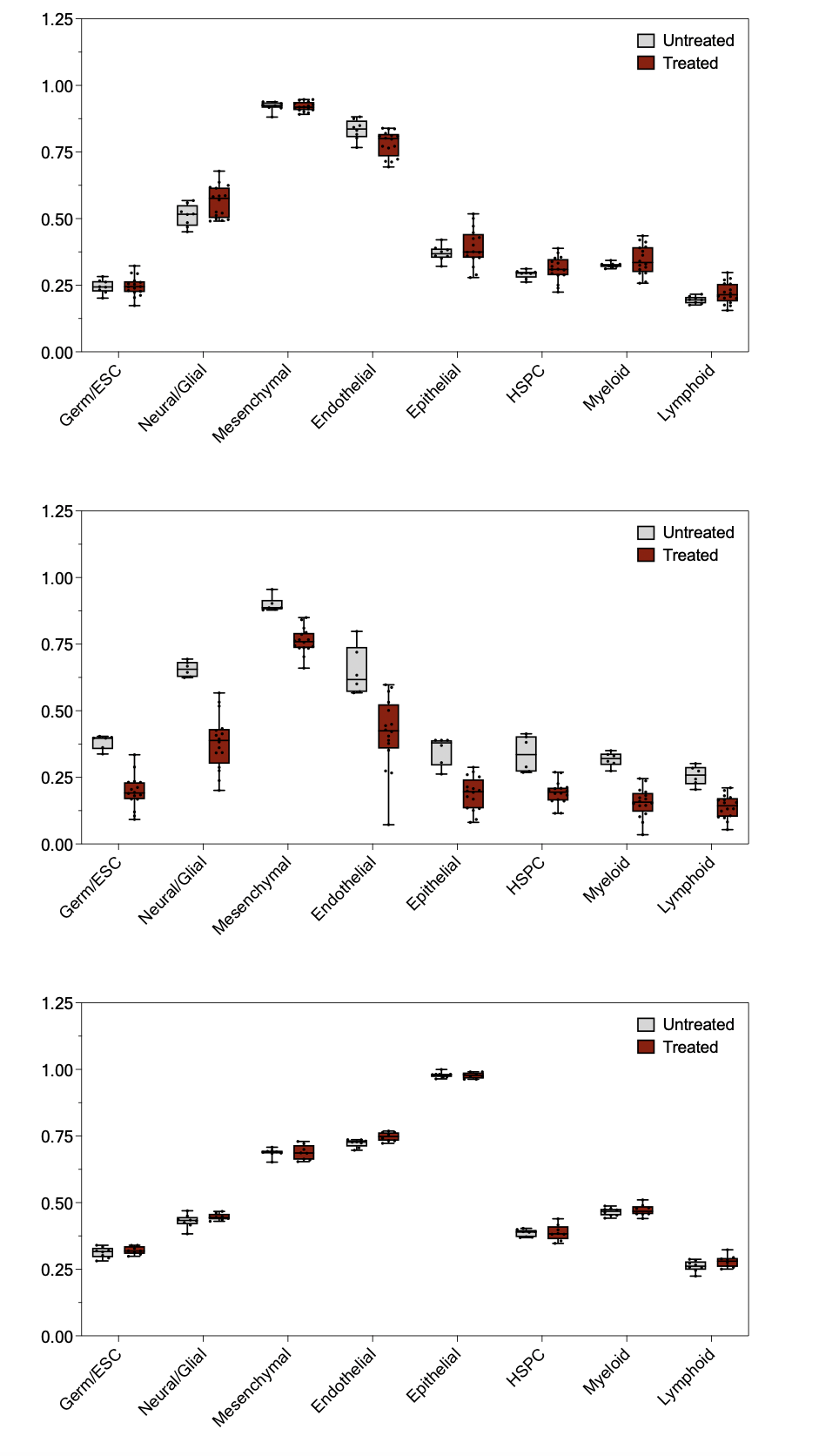


U118MG

HuO9

HCC1954

CANCER CELLS


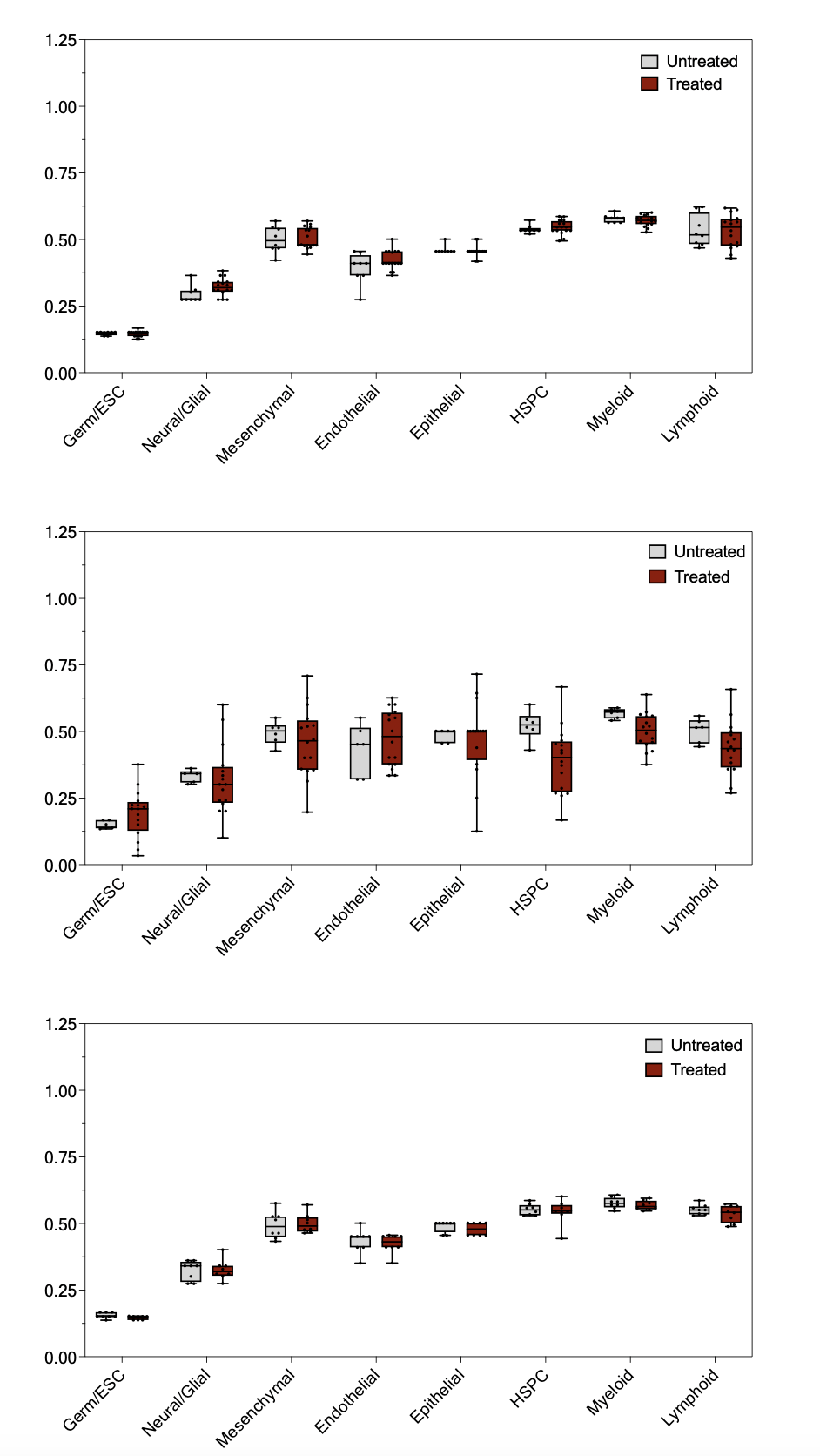


STROMAL CELLS

Supplementary Figure 11.

Relative cell characterization of individual tumor samples using Syllogist. Overall, 43 cell types were analyzed and relative expression was averaged into seven summary cell types as shown (Germ/ESC, Neural/Glial, Mesenchymal, Endothelial, Epithelial, HSPC, Myeloid, Lymphoid). Normalized relative expression of cell types is presented on a scale of 0 (underrepresented cell type, not present in sample) to 1 (overrepresented cell type, present in sample). U118MG and HuO9 cancer cells were characterized as mesenchymal phenotypes, whereas HCC1954 cancer cells were majority epithelial (left plots), in line with relative LRRC15 cancer cell expression between the three models. In stromal cells, no significant cell phenotype was observed in any of the three models (right plots).

**a** **b**
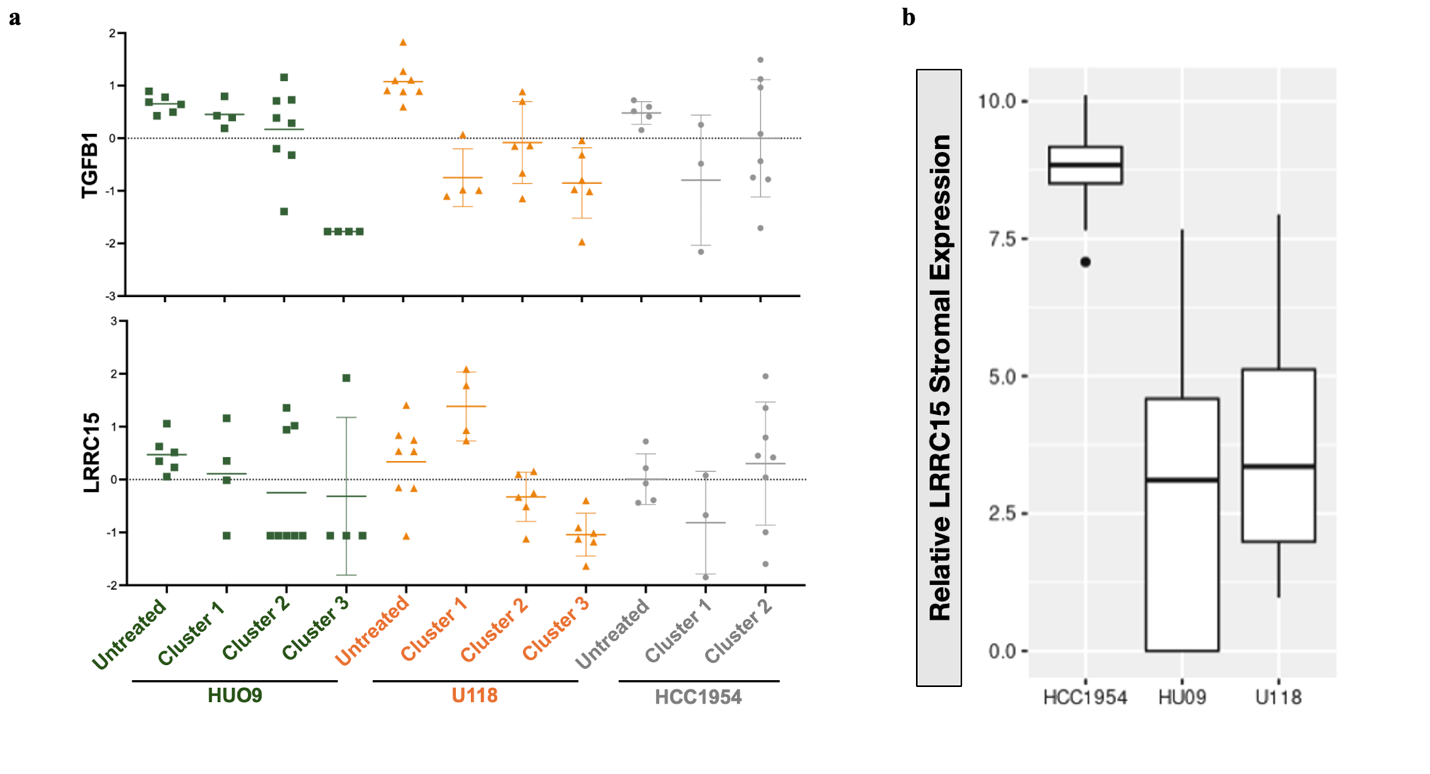


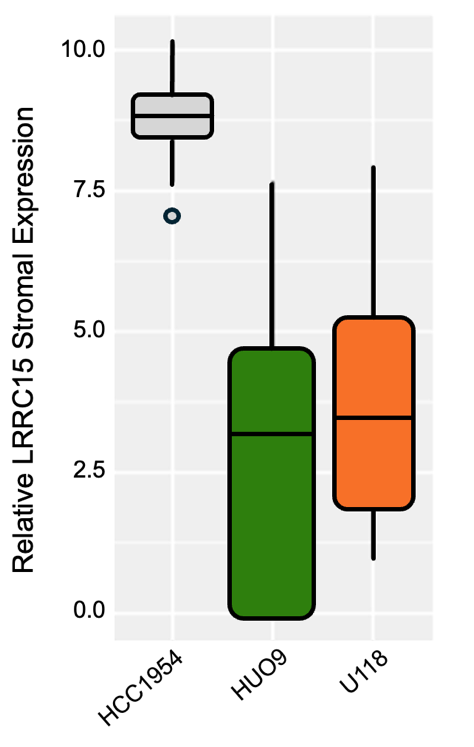


Supplementary Figure 12.

Stromal cell expression of *Lrrc15* and *Tgfb1*. **a.** Transcript data from clustered (Supplementary Figure 10) tumor stroma of *Lrrc15* (top) and *Tgfb1* (bottom) relative expression, represented by box-and-whisker plots. HuO9 transcripts are plotted in green (left), U118MG in orange (middle), and HCC1954 in grey (right). In HuO9 and U118MG stromal cells, expression of *Tgfb1* is significantly reduced in cluster 3 (p<0.05). Across tumor models, changes in *Lrrc15* transcript expression were not significant between treated and untreated samples. **b.** Comparison of relative baseline transcript expression of *Lrrc15* in tumor stroma, comparing HCC1954 (right), HuO9 (middle) and U118MG (left). In HCC1954 tumors, median stromal *Lrrc15* expression is high (8.91) compared to moderate expression in HuO9 (3.02) and U118MG (3.48).


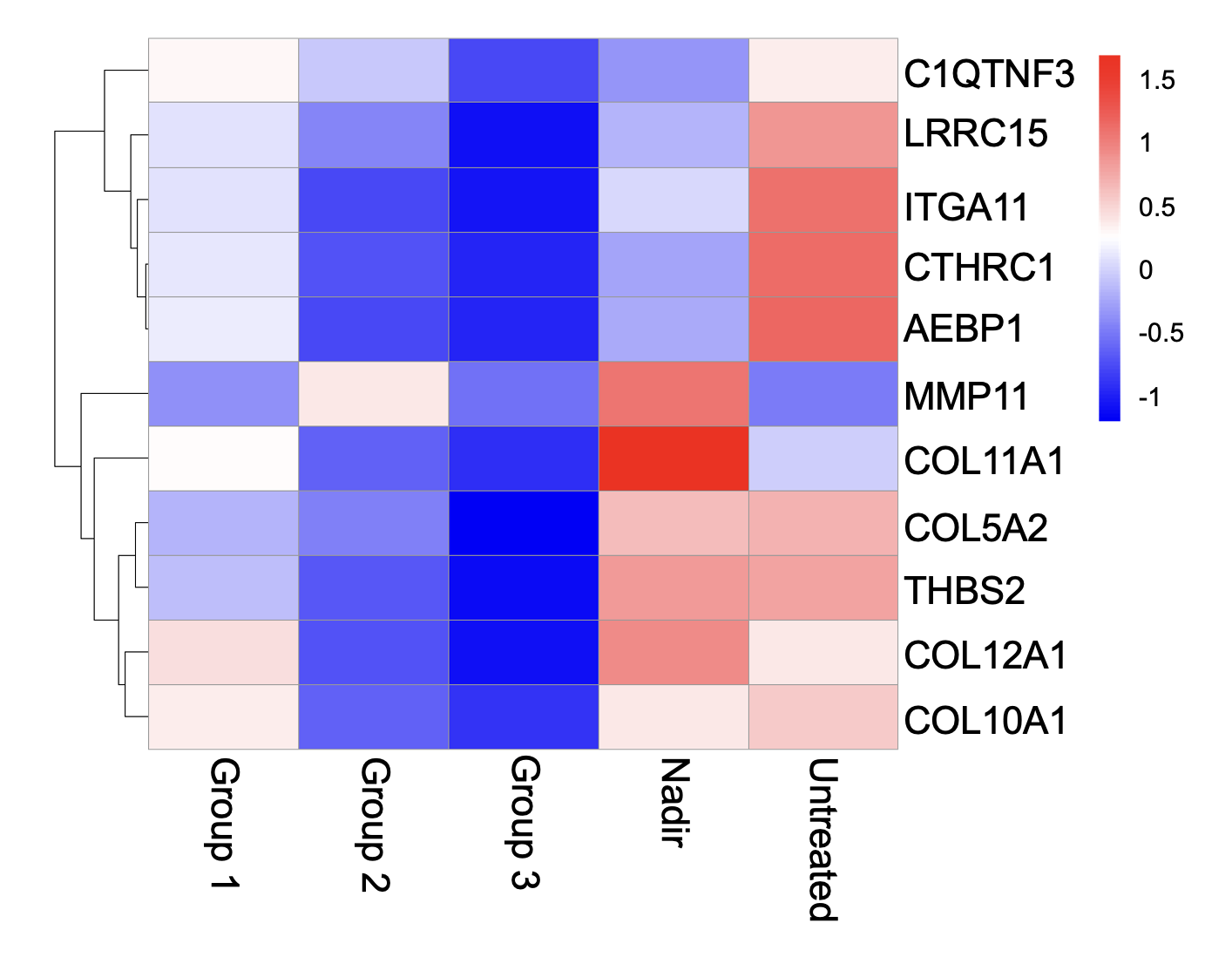


Supplementary Figure 13.

Transcript data from clustered (Supplementary Figure 10) tumors (90-120d post-treatment) and tumors harvested at nadir (30d post-treatment) show decreased expression of the LRRC15+ TGFβ signature. U118MG tumors lose expression of the LRRC15+ TGFβ signature after [^177^Lu]Lu-DUNP19 treatment (red = high, blue = low expression), with expression of the LRRC15+ TGFβ signature reaching an intermediate phenotype at nadir.


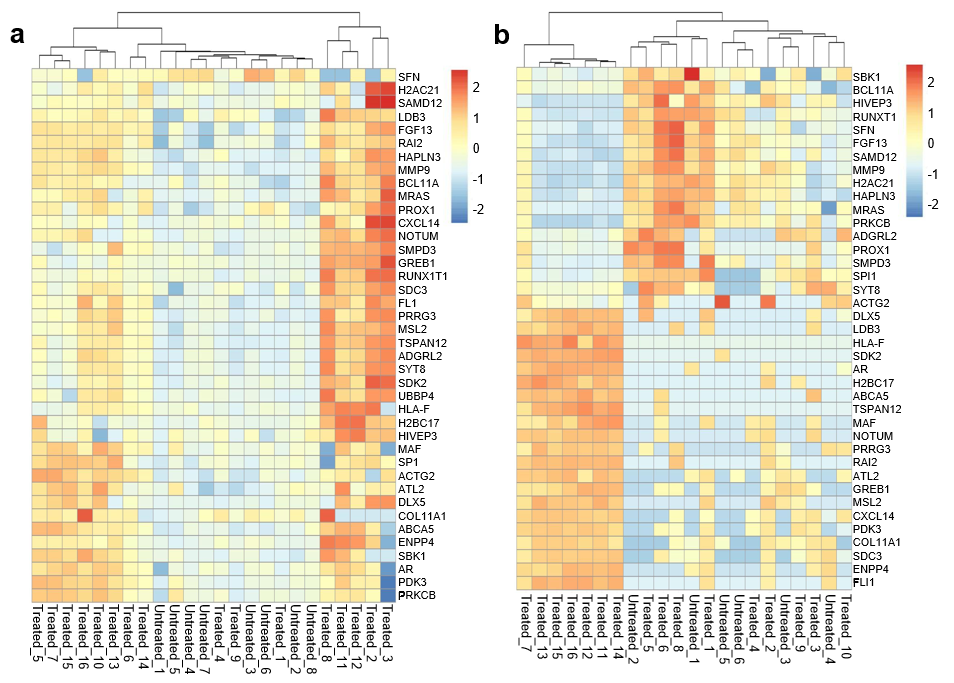


Supplementary Figure 14.

Transcript data overlapping differentially expressed genes (40 genes, FDR < 0.05, FC > 1) in (a) U118MG and (b) HuO9 cancer cells after [177Lu]Lu-DUNP19 treatment were plotted for visualization of [177Lu]Lu-DUNP19-induced changes. Relative expression per gene was plotted to indicate up- (in red) or downregulated (blue) genes by Z-score normalization.

**Supplementary Tables**

Supplementary Table 1.

Biodistribution of [^177^Lu]Lu-DUNP19 in male BALB/c-nu/nu mice bearing U118-MG glioblastoma xenografts, 24, 48 and 72 h after intravenous injection. The measured radioactivity of different organs is expressed as %IA/g, and presented as an average value from 3-4 animals ± SD. * *GI tract uptake is presented as %IA only.*

|  | **24 h** | **48 h** | **72 h** |
| --- | --- | --- | --- |
| **Blood** | 9.63±0.77 | 7.57±1.39 | 5.75±0.73 |
| **Salivary gland** | 1.74±0.35 | 1.92±0.14 | 1.89±0.21 |
| **Heart** | 2.40±0.50 | 2.10±0.55 | 1.74±0.14 |
| **Lung** | 4.60±0.77 | 4.21±0.88 | 3.34±0.64 |
| **Liver** | 6.22±3.00 | 4.79±0.74 | 6.17±1.19 |
| **Spleen** | 4.61±1.83 | 4.79±1.22 | 4.03±1.13 |
| **Stomach** | 0.71±0.27 | 0.56±0.29 | 0.71±0.15 |
| **Kidney** | 3.82±0.54 | 2.92±0.37 | 2.40±0.24 |
| **Tumor** | 12.04±0.92 | 13.68±2.06 | 13.30±1.08 |
| **Muscle** | 0.65±0.27 | 1.14±0.25 | 0.96±0.32 |
| **Bone** | 0.44±0.30 | 1.35±0.63 | 0.97±0.23 |
| **Brain** | 0.12±0.06 | 0.18±0.03 | 0.28±0.07 |
| **GI tract*** | 2.63±0.34 | 2.24±0.26 | 2.33±0.31 |

Supplementary Table 2.

Biodistribution of [^177^Lu]Lu-DUNP19 in male BALB/c-nu/nu mice bearing murine K7M2 osteosarcoma and human HCC1954 breast cancer xenografts after intravenous injection. The measured radioactivity of different organs is expressed as %IA/g, and presented as an average value from 3-4 animals ± SD. ** GI tract uptake is presented as %IA only.*

| K7M2*^LRRC15+^* | | | HCC1954 | |
| --- | --- | --- | --- | --- |
|  | **48 h** | **72 h** |  | **72 h** |
| **Blood** | 7.62±0.76 | 6.98±1.13 | **Blood** | 6.95±1.67 |
| **Lung** | 3.80±0.37 | 4.55±1.19 | **Lung** | 4.01±1.03 |
| **Liver** | 4.67±1.21 | 4.88±1.22 | **Liver** | 4.37±0.72 |
| **Spleen** | 3.15±0.84 | 3.04±0.82 | **Spleen** | 3.97±0.49 |
| **Stomach** | 0.81±0.11 | 0.91±0.27 | **Stomach** | 0.81±0.08 |
| **Small Intestines** | 0.99±0.04 | 1.05±0.18 |  |  |
| **Colon** | 0.72±0.14 | 0.98±0.24 |  |  |
| **Kidney** | 2.9±0.3 | 2.76±0.32 | **Kidney** | 2.77±0.35 |
| **Tumor** | 10.5±0.69 | 13.60±1.49 | **Tumor** | 11.83±2.5 |
| **Skin** | 4.88±0.52 | 6.43±1.45 | **Skin** | 6.04±1.06 |
| **Muscle** | 0.83±0.12 | 1.12±0.59 | **Muscle** | 0.95±0.36 |
| **Bone** | 1.21±0.28 | 1.17±0.40 | **Bone** | 1.16±0.12 |
| **Brain** | N/A | 0.26±0.07 | **GI tract*** | 2.29±0.31 |

Supplementary Table 3.

Gene set enrichment analysis of HuO9, U118MG, and HCC1954 cancer cells.

| **PATHWAY** | **Pval** | **Padj** | **Enrich Score** | **Norm Enrich Score** |
| --- | --- | --- | --- | --- |
| **HUO9** |  |  |  |  |
| HALLMARK E2F TARGETS | 0.000 | 0.001 | -0.380 | -1.752 |
| HALLMARK CHOLESTEROL HOMEOSTASIS | 0.001 | 0.015 | -0.453 | -1.700 |
| HALLMARK FATTY ACID METABOLISM | 0.002 | 0.015 | -0.392 | -1.643 |
| HALLMARK OXIDATIVE PHOSPHORYLATION | 0.001 | 0.015 | -0.351 | -1.595 |
| HALLMARK GLYCOLYSIS | 0.001 | 0.015 | -0.362 | -1.584 |
| **U118MG** |  |  |  |  |
| HALLMARK MYC TARGETS | 0.000 | 0.000 | -0.583 | -2.406 |
| HALLMARK MYCOGENESIS | 0.000 | 0.000 | 0.664 | 1.745 |
| HALLMARK TNFA SIGNALING VIA NFKB | 0.000 | 0.000 | 0.623 | 1.637 |
| HALLMARK KRAS SIGNALING DN | 0.000 | 0.000 | 0.666 | 1.698 |
| HALLMARK KRAS SIGNALING UP | 0.000 | 0.000 | 0.613 | 1.607 |
| **HCC1954** |  |  |  |  |
| HALLMARK INTERFERON GAMMA RESPONSE | 0.000 | 0.000 | -0.620 | -2.186 |
| HALLMARK INTERFERON ALPHA RESPONSE | 0.000 | 0.000 | -0.695 | -2.286 |
| HALLMARK OXIDATIVE PHOSPHORYLATION | 0.000 | 0.000 | 0.446 | 2.063 |
| HALLMARK GLYCOLYSIS | 0.000 | 0.000 | 0.425 | 1.945 |
| HALLMARK INFLAMMATORY RESPONSE | 0.000 | 0.000 | -0.554 | -1.903 |
